# The C-terminal region of TENT5 proteins drives ER-associated mRNA polyadenylation via FNDC3 interaction

**DOI:** 10.1016/j.celrep.2026.117501

**Published:** 2026-06-04

**Authors:** Lisa Viviani, Daniel Lacidogna, Sara Pennacchio, Maria Vittoria Mengozzi, Ugo Orfanelli, Leone Giordano, Tommaso Perini, Simone Cenci, Enrico Milan

**Affiliations:** 1 Age Related Diseases Unit, Division of Genetics and Cell Biology, IRCCS San Raffaele Scientific Institute, 20132 Milan, Italy; 2 University Vita-Salute San Raffaele, 20132 Milan, Italy; 3 ENT Department, IRCCS Ospedale San Raffaele, 20132 Milan, Italy; 4 Hematology and Bone Marrow Transplantation Unit, IRCCS Ospedale San Raffaele, 20132 Milan, Italy; 5 University of Padova, Department of Biology, 35131 Padova, Italy

**Keywords:** endoplasmic reticulum, FAM46, FNDC3, multiple myeloma, polyadenylation, secretion, TENT5

## Abstract

Spatial regulation of mRNA polyadenylation emerges as a key mechanism shaping cellular function. The TENT5/FAM46 family comprises four non-canonical poly(A) polymerases that stabilize transcripts encoding ER-targeted proteins, with mutations linked to diseases of professional secretory cells. Using transcriptomic and proteomic profiling with systematic mutagenesis, we show how paralog-specific divergence drives distinct localization, interactions, and functions. TENT5D, the most ER-associated member, remodels the proteome, enhancing the expression of ER, ERGIC, Golgi, and lysosomal proteins. In contrast, TENT5B lacks ER targeting and regulates proteins involved in cell division. A member-specific C-terminal region that binds ER-transmembrane FNDC3 proteins is necessary and sufficient for ER localization. Mutations in this region of TENT5C, found in multiple myeloma, impair FNDC3 binding or stability, reducing immunoglobulin production and tumor-suppressive activity. Overall, we define a domain-encoded mechanism linking TENT5 localization to transcript selectivity and secretory output, with direct implications for compartmentalized mRNA regulation and development of RNA-based therapeutic strategies.

## Highlights

• ER-localized TENT5 proteins enhance the expression of secretory pathway components
• Subcellular localization dictates TENT5 substrate specificity among paralogs
• A divergent C-terminal region recruits TENT5 proteins to the ER via FNDC3
• Mutations in the helical and C-terminal regions disrupt ER targeting or stability

## Graphical abstract

**Figure:**
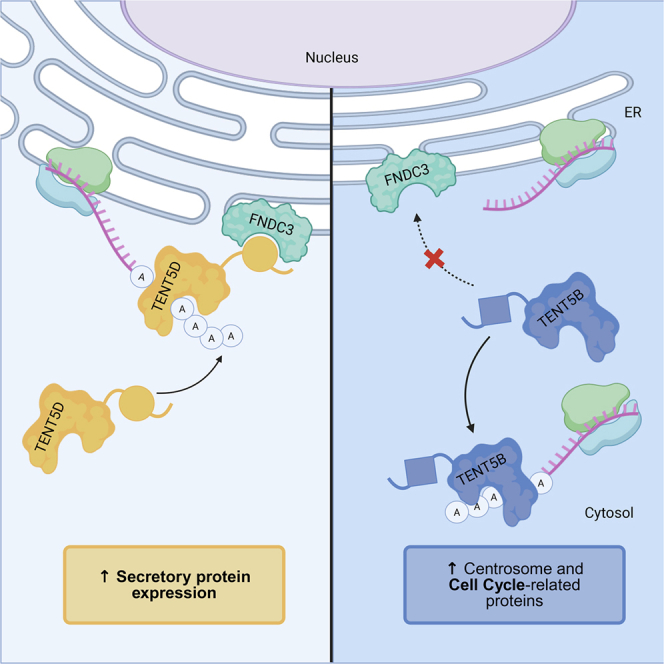

## Teaser

Viviani et al. identify the molecular determinants that recruit TENT5 poly(A) polymerases to the endoplasmic reticulum. A divergent C-terminal region mediates interaction with FNDC3 proteins, enabling ER localization and broad stabilization of transcripts encoding secretory pathway components. This spatial mechanism explains functional divergence among TENT5 paralogs.

## Introduction

Terminal nucleotidyl-transferase 5 (TENT5/FAM46) proteins constitute a conserved family of non-canonical poly(A) polymerases (ncPAPs) that have recently emerged as pivotal post-transcriptional regulators of the secretory proteome.^1^^,^^2^^,^^3^ In humans, the four TENT5 paralogs (TENT5A–D) are cytoplasmic enzymes expressed in distinct cell types, where they preferentially stabilize transcripts encoding proteins targeted to the endoplasmic reticulum (ER).^4^ This activity is crucial for the execution of tissue-specific secretory programs, as evidenced by the involvement of TENT5 mutations in a large range of seemingly heterogeneous pathologies, including osteogenesis imperfecta, multiple myeloma (MM), and male infertility.^5^^,^^6^^,^^7^^,^^8^ TENT5A, for example, promotes collagen deposition by osteoblasts, and its loss-of-function mutations are causative of a rare form of osteogenesis imperfecta.^3^^,^^5^^,^^9^ TENT5C supports antibody production by plasma cells (PCs), and it is uniquely and frequently deleted or mutated in MM.^6^^,^^10^^,^^11^ We have recently proposed that transformed PCs may inactivate TENT5C to reduce immunoglobulin (Ig) production and its associated stress and energy demands, thereby prioritizing their own growth.^12^ TENT5D, in turn, has been linked to impaired spermatid morphogenesis and rare forms of male infertility.^7^^,^^8^

Beyond these established functions, recent studies have further expanded the physiological and biomedical relevance of the TENT5 family. TENT5A has been shown to modulate immune responses to mRNA vaccines by stabilizing synthetic transcripts and protecting them from de-adenylation,^13^ while polymorphisms in TENT5C have been associated with altered insulin secretion in pancreatic β-cells, implicating a possible role in glucose homeostasis and predisposition to diabetes.^14^

Remarkably, despite lacking canonical RNA-binding domains or recognizable sequence motifs in their mRNA targets,^15^ TENT5 proteins are believed to achieve substrate specificity through spatial localization. Indeed, increasing evidence by our group and others supports a model in which TENT5 enzymes are recruited to the ER membrane where secretory transcripts are translated.^11^^,^^13^ This dynamic representation has been proposed to be mediated by TENT5 interaction with ER transmembrane proteins of the FNDC3 family, namely FNDC3A and FNDC3B.^11^^,^^13^^,^^16^ However, the molecular requirements of TENT5 recruitment to the ER membrane remain largely undefined.

Importantly, our previous study highlighted considerable heterogeneity among TENT5 proteins in terms of cellular localization, FNDC3 binding affinity, protein stability, and impact on the secretory proteome.^11^ The determinants of this variability remain unknown, as does the cellular role of TENT5B, the least ER-associated member of the family. In this study, we investigate the molecular basis dictating ER localization, FNDC3 binding, and protein stability of TENT5 family members to clarify how their domain architecture defines their ER-associated polyadenylation activity. Our findings provide more insights into the functional diversity of the TENT5 proteins and unveil molecular mechanisms orchestrating spatially regulated mRNA modifications, with potential implications for disease mechanisms, immune regulation, and the development of RNA-based therapeutics.

## Results

### TENT5 proteins differ in subcellular localization, interaction with FNDC3 proteins, and impact on the secretory pathway

**Figure 1:**
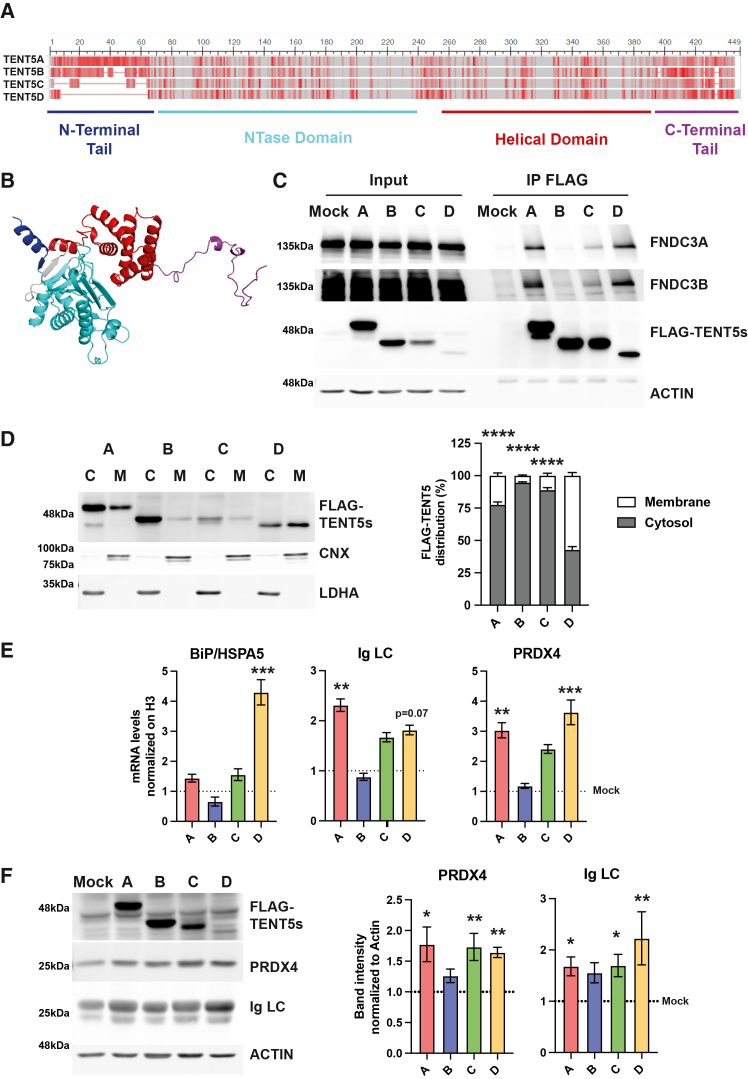
TENT5 proteins differ in their interaction with FNDC3 proteins, cellular localization, and effects on ER-targeted proteins (A) TENT5 proteins alignment using Blastp (blast.ncbi.nlm.nih.gov/Blast.cgi), in gray conserved amino acids, while red highlights variability. (B) AlphaFold-predicted model of TENT5C (AF-Q5VWP2-F1-v4), visualized in PyMOL. The structure is colored according to the domains indicated in (A). Image created with PyMOL v3.1.6.1 (Schrödinger, LLC). (C) Co-immunoprecipitation of FNDC3 proteins with FLAG-TENT5s in LP1 cells. (D) Immunoblot analysis for the cellular distribution of TENT5 proteins in LP1 cells. Left: representative blot (C= cytosol, M = membrane); right: quantification of FLAG-TENT5 abundance in the cytosolic and membrane-associated fractions (mean ± SEM; Ordinary two-way ANOVA with Dunnett’s multiple comparison vs. TENT5D infected cells, *n* = 7, ^∗∗∗∗^*p* < 0.0001). (E) qRT-PCR analysis of the indicated mRNAs in LP1 cells expressing TENT5 paralogs relative to mock-infected cells (mean ± SEM normalized to H3 mRNA; Kruskal Wallis test with Dunn’s multiple comparison vs. Mock, *n* = 6, ^∗∗^*p* < 0.01; ^∗∗∗^*p* < 0.001). (F) Immunoblot analysis of the indicated proteins in LP1 cells expressing TENT5 proteins. Left: representative blots, right: quantification of protein band intensity normalized to actin, expressed as relative to mock-infected cells (mean ± SEM, Kruskal Wallis test with Dunn’s multiple comparison vs. Mock, *n* = 5, ^∗^*p* < 0.05; ^∗∗^*p* < 0.01).

All TENT5 members share two conserved core domains: a catalytic nucleotidyl-transferase (NTase) domain and a C-terminal helical domain (HD), whose function remains poorly understood (Figures 1A and 1B). In contrast, their N-terminal and C-terminal regions vary substantially in both length and structure, accounting for the differences in overall size across family members. These terminal extensions are less structurally defined and are not predicted to form stable domains. Despite high sequence identity (spanning from 73% between TENT5A and TENT5C and 52% between TENT5B and TENT5D; Figure 1A), our previous work showed striking differences among TENT5 paralogs in terms of intracellular localization, protein stability, and effects on the secretory pathway.^11^ In this study, we leveraged these intrinsic differences to dissect the molecular features underpinning spatially regulated mRNA polyadenylation. To this end, we expressed distinct TENT5 paralogs, mutants, and chimeras in LP1 MM cells, which naturally lack TENT5C,^17^ the member normally expressed in PCs. This system enabled a rapid and quantitative comparison of the impact of each TENT5 variant on secretory output, using Ig light chain (LC) production as a functional readout.^11^^,^^12^

Among all members, the testis-specific TENT5D displayed the strongest interaction with FNDC3 proteins and predominantly localized to the ER (Figures 1C and 1D). Conversely, TENT5B showed little to no FNDC3 binding and was mostly excluded from membrane fractions. To complement the biochemical fractionation analysis, we also examined the subcellular localization of TENT5 paralogs by confocal immunofluorescence microscopy. Co-localization analysis with the ER marker PDI confirmed the ER localization of FNDC3 interacting paralogs, whereas TENT5B displayed a largely cytosolic distribution (Figure S1). This differential localization correlated with distinct functional effects on the expression of ER-targeted proteins (Figures 1E and 1F). Consistent with its ER association, TENT5D induced the most robust increase in Ig LC production, despite its previously reported lower protein stability.^11^ On the contrary, TENT5B, although catalytically active and more stable,^2^^,^^11^^,^^18^ lacking the specificity conferred by ER localization, elicited only smaller increases in Ig LC levels, likely due to the high abundance of this mRNA in MM cells, but had minimal effects on ER-resident mRNAs and proteins (Figures 1E and 1F). TENT5A and TENT5C exhibited intermediate phenotypes in terms of localization, FNDC3 affinity, and effects on secretory mRNA and protein levels (Figures 1C–1F). These findings support a model in which FNDC3-mediated anchoring and ER-localization drive the capacity of TENT5 proteins to enhance secretory gene translation. This mechanistic link may also explain why FNDC3-interacting TENT5 members are associated with diseases of secretory cells, unlike TENT5B, whose physiological role remains elusive. The distinct localization and functional features of TENT5 paralogs are summarized in Table 1.

**Table 1:** Summary of TENT5 paralog features

	Tissue	Main known targets	Cellular localization	Associated diseases
TENT5A	bone marrow, salivary glands (Human Protein Atlas), osteoblasts^3^	COL1A1, COL1A2, Sparc, SerpinF1^3^	cytosol and ER	osteogenesis imperfecta^5^
TENT5B	esophagus and skin (Human Protein Atlas), stem cells^18^	WNT5A, NANOG, RICTOR, SKP2^18^	cytosol	unknown
TENT5C	bone marrow, pancreas, testis (Human Protein Atlas), PCs^1^	immunoglobulin and other ER-targeted mRNAs^1^^,^^10^	cytosol and ER	multiple myeloma^6^
TENT5D	testis (Human Protein Atlas)	RNASET2, Adam26a, Cldn34c4^2^	ER	male sterility^7^^,^^8^

### Cellular localization dictates the functional impact of TENT5 proteins

**Figure 2:**
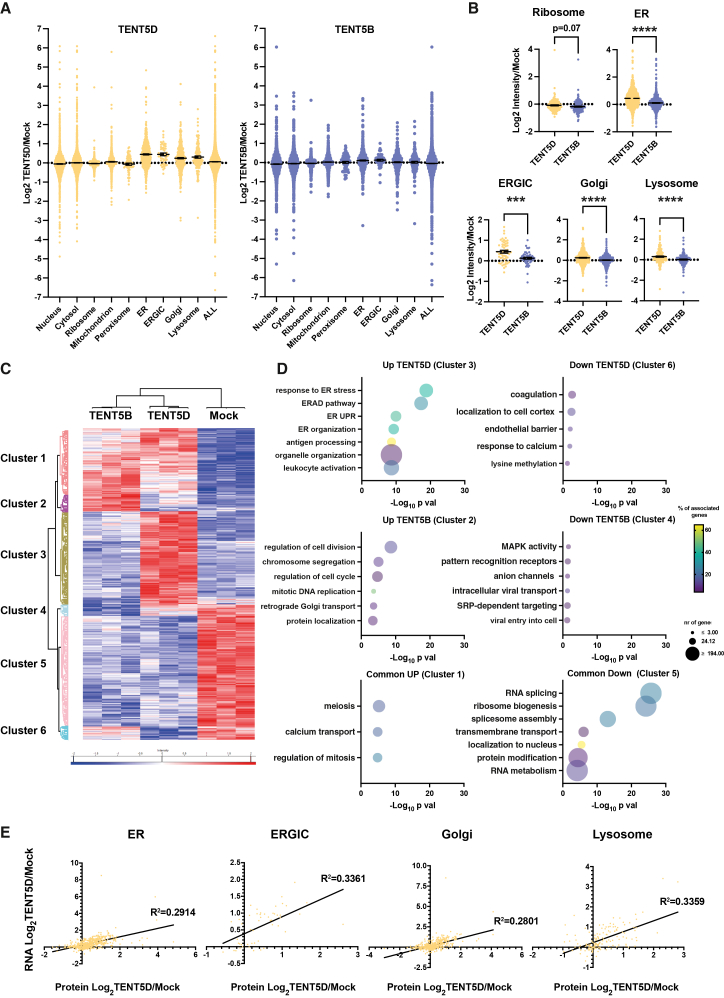
Cytosolic TENT5B has minor effects on the secretory proteome (A) Proteome changes in LP1 cells overexpressing TENT5D (left) or TENT5B (right) compared to mock-infected cells by label-free LC-MS/MS analysis. The 6910 proteins quantified were grouped by the indicated gene ontology (GO) categories (Nucleus GO:0005634; Cytosol GO:0005829; Mitochondria GO:0005739; Ribosome GO:0005840; Peroxisome GO:0005777, ER GO:0005783; ERGIC GO:0033116; Golgi GO:0005794; Lysosome GO:0005764; average ratios ±SEM). (B) Fold change relative to mock-infected cells of quantified proteins belonging to the indicated cell compartments (average ratios ±SEM, unpaired *t* test, ^∗∗∗^*p* < 0.001; ^∗∗∗∗^*p* < 0.0001). (C) Heatmap and hierarchical clustering of 1,897 ANOVA-significant DEPs with FDR<0.01 (in blue: downregulated; red: upregulated). (D) Gene ontology (GO) enrichment analysis of DEPs by ClueGO showing the most significant enriched Biological Processes (BP) for each cluster. (E) Correlation analysis of effects on mRNA and protein levels, grouped by the indicated gene ontology (GO) categories, induced by TENT5D expression compared to mock-infected cells (Pearson’s R squared).

To explore the functional diversity of TENT5 paralogs, we performed label-free quantitative mass spectrometry in LP1 cells expressing the most divergent members, TENT5B and TENT5D. Proteomic profiling revealed profound differences (Data S1), as evidenced by the complete separation of TENT5B and TENT5D samples in unsupervised PCA (Figure S2A). Among the two, TENT5D triggered a pronounced proteomic remodeling, with the marked upregulation of proteins localized to the ER, ER-Golgi intermediate compartment (ERGIC), Golgi apparatus, and lysosomes, underscoring its key role in secretory pathway regulation (Figures 2A and 2B). Notably, this extensive reorganization of organelle-specific proteomes was driven by the expression of a single cytoplasmic poly(A) polymerase, highlighting the potency of localized RNA regulation in shaping cellular identity and function. In contrast, TENT5B exerted more modest effects, causing minimal changes in organelle-specific proteomes. Specifically, TENT5B led to a slight increase in ERGIC proteins and a depletion of ribosomal components relative to mock controls (Figures 2A and 2B). These observations were independently validated in HEK293T cells (Data S2), where TENT5D consistently enhanced the abundance of secretory compartment proteins, while TENT5B expression resulted in a reproducible reduction in ribosomal protein levels (Figures S2B and S2C), reinforcing the robustness of these proteomic effects across distinct cellular systems.

In total, 1,897 significantly deregulated proteins (FDR <0.01) were identified upon TENT5 expression in LP1 cells. Hierarchical clustering grouped them into six main clusters (Figure 2C and Data S1). Functional enrichment analysis for Biological Processes (ClueGO, GOterm-BP-FAT) of cluster-specific genes highlighted a strong TENT5D-driven activation of ER-related pathways, including ER-associated degradation (ERAD), unfolded protein response (UPR), and ER-to-Golgi transport (Figure 2D). This coordinated modulation suggests that ER-localized TENT5 members operate as master regulators of functional identity and ER homeostasis in secretory cells. Conversely, proteins upregulated specifically by TENT5B, or shared targets, were enriched for factors involved in cell cycle progression, cell division, and chromosome segregation (Figure 2D), consistent with previously reported roles for TENT5B in stemness and centrosome biology.^18^^,^^19^^,^^20^ Notably, genes involved in meiosis were also enriched among common targets, aligning with fertility defects observed in TENT5B and TENT5C double knockout mice and the known association of TENT5D mutations in male infertility.^2^^,^^7^

Consistent with the RNA-stabilizing activity of TENT5 proteins, few protein targets were specifically downregulated by TENT5D or TENT5B (Figure 2D). However, both paralogs robustly reduced the abundance of proteins involved in RNA metabolism and ribosome biogenesis, in line with their impact on cellular compartment composition (Figures 2A and 2B). These results reveal a substantial reprogramming of RNA-related networks by TENT5-mediated polyadenylation, pointing to a previously unappreciated regulatory axis that warrants further investigation.

To determine whether the observed proteomic remodeling reflects coordinated changes at the transcript level, we performed RNA sequencing in LP1 cells expressing TENT5D, TENT5B, or control vectors. Transcriptomic profiling revealed strong concordance with the proteomic dataset, with transcripts encoding proteins localized to the ER, ER-Golgi intermediate compartment (ERGIC), and lysosomes being consistently upregulated upon TENT5D expression but not TENT5B (Figures 2E and S2D and Data S3). Integration of RNA-seq and proteomic data supports a model in which TENT5D-mediated polyadenylation stabilizes transcripts encoding secretory pathway components.

**Figure 3:**
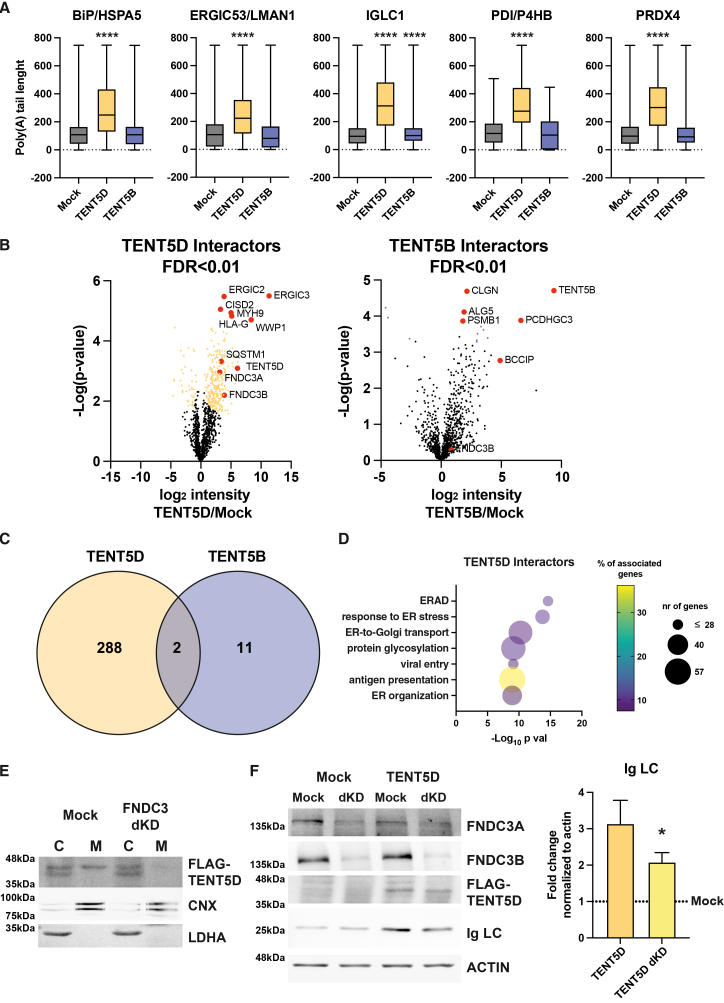
FNDC3 interaction determines spatially regulated ER-associated mRNA stabilization (A) TAIL-seq analysis of poly(A) tail length in the indicated mRNAs (HSPA5 = ENST00000324460; LMAN1 = ENST00000251047; IGLC1 = ENSG00000211675.t1; P4HB = ENST00000948310; PRDX4 = ENST00000379341) from LP1 cells expressing TENT5D, TENT5B, or control cells (median-Min to Max; Kruskal Wallis test with Dunn’s multiple comparison vs. Mock, ^∗∗∗∗^*p* < 0.0001). (B) Vulcano plot of FLAG-TENT5D (left) and FLAG-TENT5B (right) interactors identified by anti-FLAG immunoprecipitation followed by LC-MS/MS analysis (colored dots indicate significant interactors with FDR<0.01). (C) Venn diagram of TENT5D and TENT5B significant interactors with FDR<0.01. (D) Gene ontology (GO) enrichment analysis of TENT5D significant interactors with FDR<0.01 by ClueGO, showing the most significant enriched Biological Processes (BP). (E) Immunoblot analysis of the cellular distribution of FLAG-TENT5D in control and double FNDC3 knockdown LP1 cells. (F) Immunoblot analysis of the indicated proteins in control or FNDC3-silenced LP1 cells. Left: representative blots; right: quantification of Ig LC band intensity normalized to actin, expressed as relative to corresponding LP1 cells not expressing FLAG-TENT5D (mean ± SEM, ratio paired *t* test, *n* = 6, ^∗^*p* < 0.05).

To directly assess poly(A) tail regulation, we next performed TAIL-seq analysis. These experiments revealed that TENT5D expression leads to a significant elongation of poly(A) tails in transcripts encoding secretory pathway components, including ER-associated proteins and Ig chains, consistent with transcriptomic and proteomic changes (Figure 3A and Data S4). In contrast, TENT5B had a minimal global impact on poly(A) tail length, with only 132 transcripts showing significant elongation, compared with 1,616 transcripts significantly elongated by TENT5D. Accordingly, the median poly(A) tail length increased from ∼105 to ∼160 nucleotides upon TENT5D expression, whereas TENT5B produced negligible global changes (Data S4).

Further supporting the functional divergence revealed by our proteome analysis, interactome profiling identified distinct binding partners for the two paralogs (Figures 3B and 3C and Data S5). TENT5D interacted with both FNDC3 proteins and multiple ER- and ERGIC-associated proteins. Moreover, we confirmed its interaction with the autophagic receptor p62/SQSTM1, previously uncovered by us,^11^ and with several E3 ligases (i.e., WWP1, WWP2, RNF149, RNF13, STUB1, and ITCH), which may regulate its degradation and protein stability. ClueGO analysis of the 288 significant TENT5D interactors (FDR <0.01) showed strong enrichment for ERAD, UPR, ER organization, and ER-to-Golgi transport proteins, mirroring the proteomic effects observed in whole-cell lysates (Figure 3D). In contrast, TENT5B displayed a limited interactome, sharing only two proteins with TENT5D (ALG5 and GUSB), further emphasizing their divergent regulatory profiles (Figure 3C). Notably, the TENT5B interactome included a previously identified partner: the proposed inhibitor BCCIP (FDR <0.05),^10^^,^^21^ which was absent from the TENT5D interactome.

The distinct capacity of TENT5D to bind ER-transmembrane FNDC3 proteins likely determines its broad remodeling effect on the secretory pathway, with FNDC3s acting as molecular anchors that direct TENT5-catalytic activity at the ER interface. To functionally validate the role of FNDC3s in our model, we generated a double knockdown (dKD) of FNDC3A and FNDC3B in LP1 cells. Genetic silencing of both family members impaired TENT5D localization at the ER membrane and significantly reduced its ability to enhance Ig LC production (Figures 3E and 3F), directly linking subcellular distribution to functional output. Together, these findings demonstrate that the localization of TENT5 proteins, shaped by specific protein-protein interactions, dictates their functional impact on cellular proteome, highlighting spatially regulated polyadenylation as a key layer of post-transcriptional control in mammalian cells.

To further support the physiological relevance of the TENT5–FNDC3 axis in professional secretory cells, we analyzed the expression of TENT5C, the paralog predominantly expressed in PCs, together with FNDC3 proteins during PC differentiation. Purified murine splenic B cells were stimulated with LPS to induce differentiation into antibody-secreting PCs. During this process, we observed a coordinated upregulation of TENT5C together with FNDC3A and FNDC3B, consistent with the activation of ER-associated secretory programs in differentiating PCs (Figures S3A and S3B). These observations support the notion that the TENT5–FNDC3 axis is physiologically engaged during PC differentiation, a cellular context characterized by extreme secretory activity.

To further validate the interaction between TENT5 proteins and FNDC3 in primary human cells, we analyzed PCs isolated from human tonsils. Co-immunoprecipitation experiments revealed that endogenous TENT5C interacts with FNDC3B in human PCs (Figure S3C). In addition, biochemical fractionation experiments in patient-derived MM cells showed that a fraction of endogenous TENT5C localizes to ER-enriched membrane fractions (Figure S3D). Together, these findings support the physiological relevance of the TENT5–FNDC3 interaction in both normal and malignant PCs.

### The TENT5 C-terminal region determines ER localization and FNDC3 interaction

To dissect the structural determinants underlying the functional specificity of TENT5 family members, we analyzed and manipulated their domain architecture. The most prominent structural differences among TENT5s lie in their N-terminal regions upstream of the NTase domain, which is nearly absent in TENT5D. We hypothesized that the lack of an extended N-terminal tail in the most ER-active paralog might reduce steric hindrance, thereby facilitating stronger interaction with FNDC3 proteins. To test this, we generated a TENT5B mutant lacking the first 48 amino acids and assessed its subcellular localization, FNDC3 binding, and impact on ER-associated mRNAs and protein expression. However, the deletion of the N-terminal segment did not enhance TENT5B’s ER localization, FNDC3 interaction, or the expression of ER-targeted mRNAs and proteins (Figures S4A–S4C). These results indicate that the N-terminal region is not a major determinant of ER targeting or FNDC3 binding.

To pinpoint the region responsible for ER localization, we thus generated a series of N- and C-terminal truncated mutants of TENT5D (Figure S4D). Notably, the deletion of the C-terminal tail and either part (I289×) or the entirety (D210×) of the HD domain abolished both FNDC3 interaction and ER membrane localization (Figures S4D and S4E). This mislocalization was accompanied by a marked reduction in the TENT5D-mediated upregulation of ER proteins Ig LC production, supporting the notion that subcellular localization determines functional specificity (Figure S4F). In contrast, the deletion of part or all of the NTase catalytic domain (Δ114 and Δ209) impaired, but did not eliminate, FNDC3 binding, allowing partial ER membrane localization (Figures S4D and S4E). As expected, despite correct subcellular localization, these ΔNTase mutants failed to promote secretory cargo expression due to loss of catalytic activity (Figure S4F). Taken together, these results demonstrate that the C-terminal region of TENT5D is essential for both ER targeting and FNDC3 interaction, whereas the catalytic domain is required for function but not localization.

**Figure 4:**
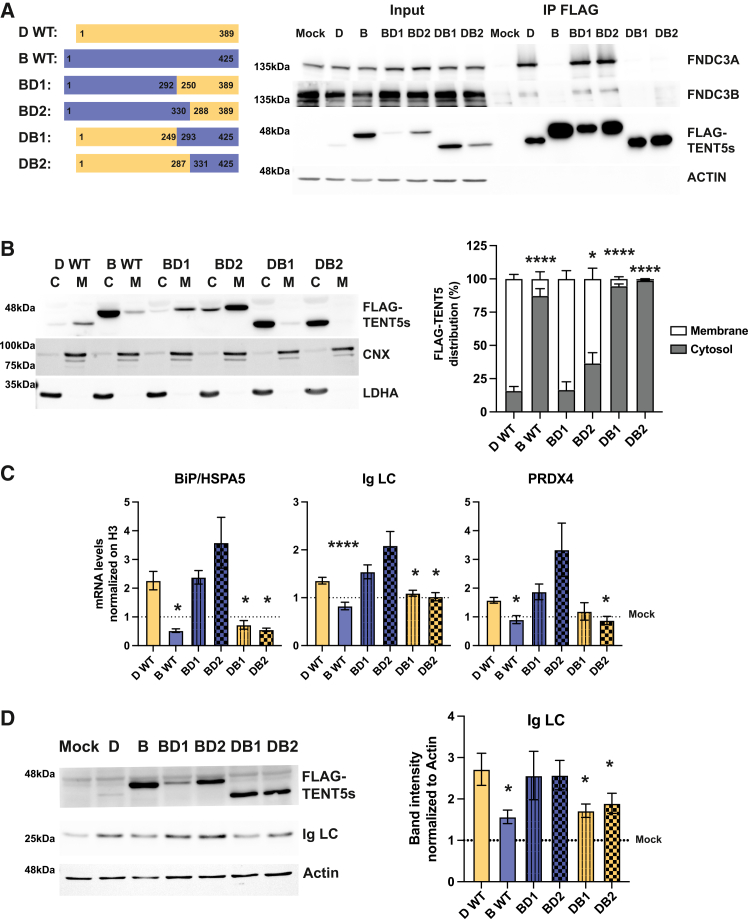
The C-terminal part of TENT5 proteins determines their association with the ER-membrane (A) Co-immunoprecipitation of FNDC3 proteins with FLAG-TENT5D/TENT5B chimeras in LP1 cells. Left: schematic representations of TENT5D/TENT5B chimeras, right: Representative immunoblots. (B) Immunoblot analysis of the cellular distribution of TENT5D/TENT5B chimeras in LP1 cells. Top: representative blot; bottom: quantification of FLAG-TENT5 abundance in the cytosolic and membrane-associated fractions (mean ± SEM; Ordinary two-way ANOVA with Dunnett’s multiple comparison vs. TENT5D-WT infected cells, *n* = 6, ^∗^*p* < 0.05; ^∗∗∗∗^*p* < 0.0001). (C) qRT-PCR analysis of the indicated mRNAs in LP1 cells expressing TENT5D/TENT5B chimeras (mean ± SEM normalized to H3 mRNA; Repeated Measure One-Way ANOVA with Dunnett’s multiple comparison vs. TENT5D-WT, *n* = 5, ^∗^*p* < 0.05; ^∗∗∗∗^*p* < 0.0001). (D) Immunoblot analysis of the indicated proteins in LP1 cells expressing TENT5D/TENT5B chimeras. Top: representative blots, bottom: quantification of Ig light chain band intensity normalized to actin, expressed as relative to mock-infected cells (mean ± SEM; Repeated Measure One-Way ANOVA with Dunnett’s multiple comparison vs. TENT5D-WT, *n* = 7, ^∗^*p* < 0.05).

To directly test the role of the C-terminal region, we generated domain-swapped chimeric mutants between TENT5B and TENT5D, exchanging the terminal portion of the HD domain between the two proteins (BD1: TENT5B 1–292/TENT5D 250–389; BD2: TENT5B 1–330/TENT5D 288–389; DB1: TENT5D 1–249/TENT5B 293–425; DB2: TENT5D 1–287/TENT5B 331–425; Figure 4A). These mutants revealed that the C-terminal region of TENT5D, starting from residue 288, is necessary and sufficient to confer FNDC3 interaction, ER localization, and increased levels of secretory mRNAs and Ig LC production (Figures 4B–4D). Strikingly, TENT5B chimeras harboring the TENT5D C-terminal segment acquired the ability to localize to the ER, bind FNDC3s, and promote secretory output, effectively mimicking wild-type TENT5D. Conversely, TENT5D chimeras carrying the C-terminal region of TENT5B lost these key features, exhibiting reduced ER localization and FNDC3 interaction, and lower Ig LC production. These findings reveal the functional importance of the C-terminal region in conferring secretory-specific properties.

**Figure 5:**
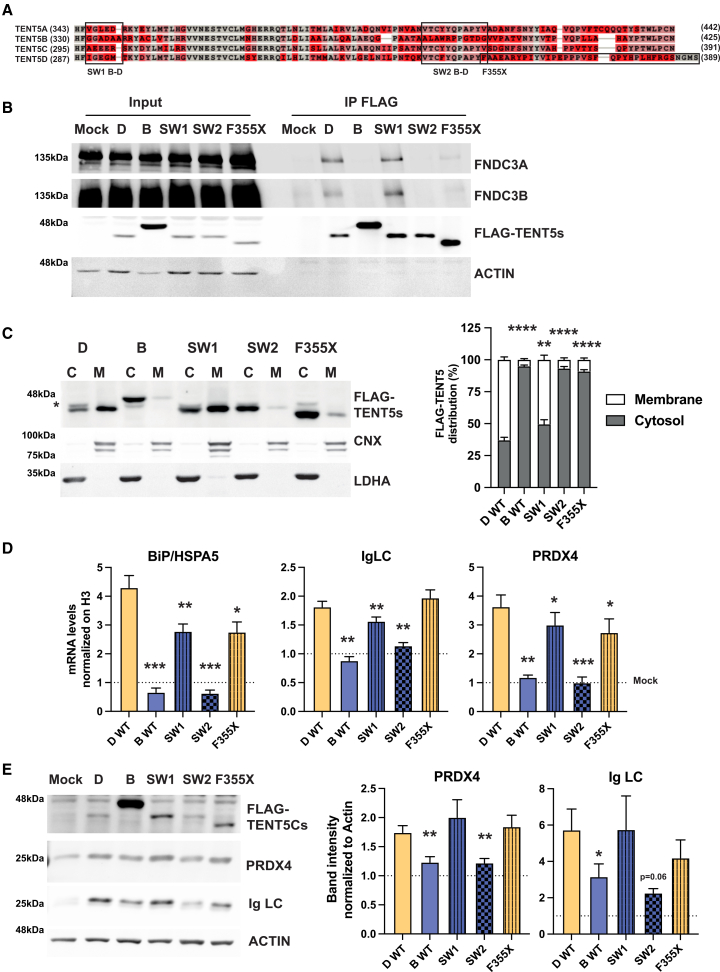
The C-terminal region mediates the interaction with FNDC3 proteins (A) TENT5 C-terminal tails alignment using Blastp (blast.ncbi.nlm.nih.gov/Blast.cgi), in gray conserved amino acids, while red highlights variability. Black boxes indicate generated mutants. (B) Co-immunoprecipitation of FNDC3 proteins with FLAG-TENT5D or its C-terminal tail variants in LP1 cells. (C) Immunoblot analysis of the cellular distribution of TENT5D and its C-terminal tail variants in LP1 cells. Left: representative blot (∗ = aspecific band); right: quantification of FLAG-TENT5 abundance in the cytosolic and membrane-associated fractions (mean ± SEM; Ordinary two-way ANOVA with Dunnett’s multiple comparison vs. TENT5D-WT infected cells, *n* = 4, ^∗∗^*p* < 0.01, ^∗∗∗∗^*p* < 0.0001). (D) qRT-PCR analysis of the indicated mRNAs in LP1 cells expressing TENT5B, TENT5D, and its C-terminal tail variants (mean ± SEM normalized to H3 mRNA; repeated measure one-way ANOVA with Dunnett’s multiple comparison vs. TENT5D-WT, *n* = 6, ^∗^*p* < 0.05; ^∗∗^*p* < 0.01 and ^∗∗∗^*p* < 0.001). (E) Immunoblot analysis of the indicated proteins in LP1 cells expressing TENT5B, TENT5D, and its C-terminal tail variants. Left: representative blots, right: quantification of protein band intensity normalized to actin, expressed as relative to mock-infected cells (mean ± SEM, Repeated measure One-Way ANOVA with Dunnett’s multiple comparisons vs. TENT5D-WT, *n* = 5, ^∗^*p* < 0.05 and ^∗∗^*p* < 0.01).

To further define the sequence elements mediating FNDC3 binding, we compared the C-terminal sequences of TENT5 paralogs. Two divergent regions, designated SW1 and SW2, differ from TENT5B and the ER-localized members (Figure 5A). In addition, the last 35 amino acids of TENT5D (355–389) are highly divergent within the family. To assess their functional relevance, we generated TENT5D/TENT5B chimeras in which either SW1 or SW2 from TENT5B was introduced into the TENT5D backbone and a truncated mutant of TENT5D losing its specific C-terminal tail: F355×. While the SW1 swap had a negligible effect, the substitution of the SW2 region completely abolished FNDC3 binding and ER targeting (Figures 5Bb and 5C), indicating that the SW2 region is essential for FNDC3 interaction and likely drives the functional divergence among TENT5 proteins. Truncation of the last 35 amino acids of TENT5D (F355×) led to a partial phenotype, with reduced FNDC3 binding and effects on secretory mRNAs and proteins (Figures 5B–5E), suggesting that while this region is not the primary binding interface, it contributes to enhanced interaction and functional output. Confocal immunofluorescence microscopy further confirmed the cytosolic redistribution of the SW2 mutant of TENT5D (Figure S5), providing complementary evidence supporting the fractionation results.

### MM mutations in the TENT5C C-terminal region impair ER localization and protein stability

We next investigated the functional impact of mutations in the HD and in the C-terminal tail of TENT5C previously identified in patients with MM.^6^ Our focus was on two mutation-enriched regions within the HD (266–269 and 291–292), and non-sense mutations within the identified FNDC3 binding region.

**Figure 6:**
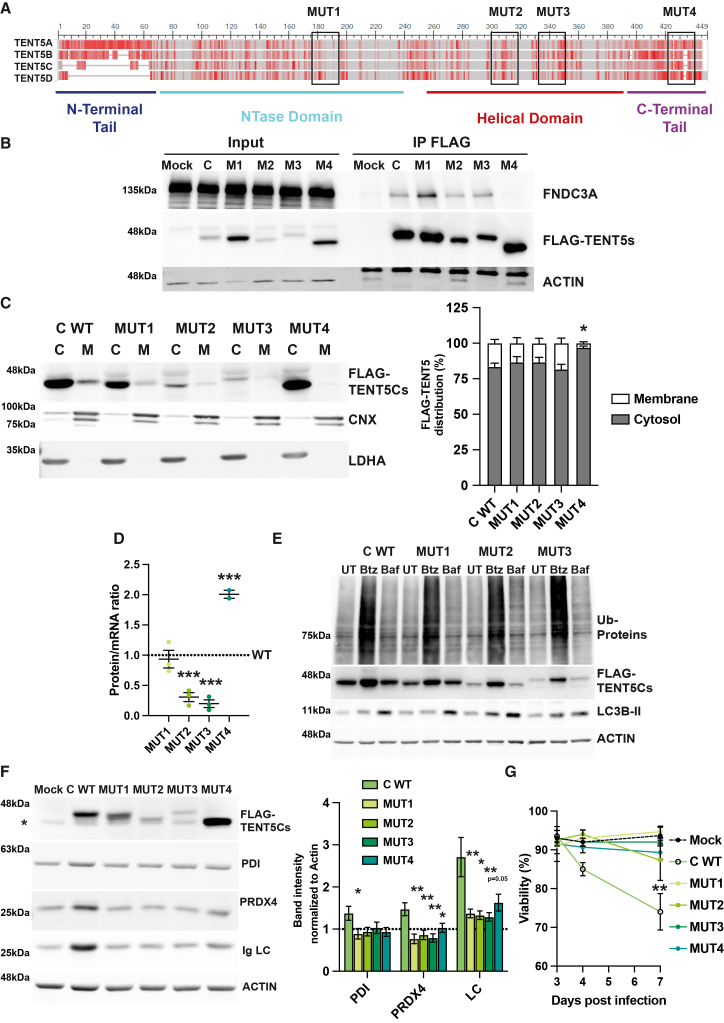
Multiple myeloma mutations in the helical domain affect TENT5C protein stability (A) TENT5s alignment using Blastp (blast.ncbi.nlm.nih.gov/Blast.cgi), in gray conserved amino acids, while red highlights variability. Black boxes indicate mutation positions. (B) Co-immunoprecipitation of FNDC3 proteins with FLAG-TENT5C or its MM-derived mutants in LP1 cells. (C) Immunoblot analysis of the cellular distribution of TENT5C and its MM-derived mutants in LP1 cells. Left: representative blot; right: quantification of FLAG-TENT5 abundance in the cytosolic and membrane-associated fractions (mean ± SEM; ordinary two-way ANOVA with Dunnett’s multiple comparison vs. TENT5C-WT infected cells, *n* = 5, ^∗^*p* < 0.05). (D) Ratio between protein intensity measured by immunoblot and mRNA levels measured by RT-qPCR for TENT5C and its mutants in LP1 cells (mean ± SEM, ordinary One-Way ANOVA with Dunnett’s multiple comparisons vs. TENT5C-WT, *n* = 3, ^∗∗∗^*p* < 0.001). (E) Representative immunoblots of the indicated proteins in LP1 cells expressing TENT5C or its mutants treated with DMSO, bortezomib (1 μM) or bafilomycin A1 (100nm) for 5 h (Quantifications are reported in Figure S6A). (F) Immunoblot analysis of the indicated proteins in LP1 cells expressing TENT5C or its mutants. Left: representative blots (^∗^ = aspecific band), right: quantification of protein band intensity normalized to actin, expressed as relative to mock-infected cells (mean ± SEM, Ordinary One-Way ANOVA with Dunnett’s multiple comparisons vs. TENT5C-WT, *n* = 6, ^∗^*p* < 0.05, ^∗∗^*p* < 0.01). (G) Cell viability of LP1 expressing TENT5C or its mutants assessed by trypan blue staining after lentiviral infection (mean ± SEM; ordinary two-way ANOVA with Dunnett’s multiple comparison vs. mock cells, *n* = 3, ^∗∗∗∗^*p* < 0.0001).

To test the impact of these alterations, we generated three additional TENT5C mutants: MUT2 (L266P, R268H, Y269C), MUT3 (Y291C, L292P), and MUT4 (Y357×) (Figure 6A). Notably, while point mutations in TENT5C are rarely repeated in MM, changes in Y291 have been recurrently identified in multiple independent sequencing studies, highlighting its potential functional relevance. As a control, we included an additional MM-derived mutant in the NTase domain (MUT1: K141T, K144E), previously shown to reduce the poly(A) polymerase activity and disrupt PLK4 binding in centrosome,^18^^,^^20^ but not involved in FNDC3 interaction. Co-immunoprecipitation and fractionation assays revealed that the SW2 truncating mutation (MUT4) completely abrogated FNDC3 binding and ER-localization, further confirming the critical role of the SW2 region (Figures 6B and 6C). In contrast, MUT2 and MUT3 retained ER-association but showed a dramatic reduction in protein stability, as evidenced by significantly lower protein-to-mRNA ratio (Figure 6D). To validate this observation, we treated cells with the proteasome inhibitor bortezomib or the autophagy inhibitor bafilomycin A1. Proteasome inhibition restored the levels of MUT2 and MUT3, indicating that these mutants are rapidly degraded via the ubiquitin-proteasome system (Figure 6E and S6A). Conversely, the NTase domain mutant (MUT1) maintained normal stability, ER localization, and FNDC3 binding (Figures 6B–6E). Despite these differences, all MM-derived mutants lost the ability to promote Ig LC production and upregulate ER-resident proteins, key features of wild-type TENT5C (Figure 6F). This impact on the secretory activity is tightly correlated with cellular toxicity, reinforcing the notion that TENT5C exerts its tumor-suppressive effects through the modulation of the secretory activity (Figure 6G).

To test whether these findings extended to other TENT5 family members, we introduced the corresponding amino acid substitutions into TENT5D to generate MUT1D (aa133–136), MUT2D (aa258–261), and MUT3D (aa283–284) (Figure 6A). Consistent with the results obtained with TENT5C, mutations in the HD led to a marked decrease in protein stability (Figures S6B–S6E). However, in contrast to the TENT5C mutants, MUT2D and MUT3D also displayed impaired FNDC3 binding and defective ER localization, suggesting that these mutations more profoundly affect the C-terminal region architecture in TENT5D. Importantly, MM-derived mutants, but not the ER-active SW1 chimera, lost all wild-type properties, including cytotoxic activity, further connecting all these aspects (Figure S6F). Taken together, our results demonstrate that MM-associated mutations in the C-terminal region of TENT5C compromise its function either by impairing ER localization or by reducing protein stability. These defects collectively diminish TENT5C’s ability to regulate the secretory pathway, strengthening the mechanistic link between antibody production and TENT5C tumor-suppressive activity in MM.

## Discussion

Our study reveals the molecular mechanisms that govern the subcellular localization and substrate specificity of TENT5 proteins, a group of ncPAPs that are critical for the selective post-transcriptional stabilization of mRNAs. The TENT5 family, appeared in Unikonta and variably expanded across vertebrates, comprises four human paralogs (TENT5A-D) characterized by conserved NTase and HDs, flanked by variable disordered terminal tails.^4^^,^^18^^,^^22^ Despite these core common features, our data highlight that differences in the evolutionarily divergent C-terminal region shape each paralog’s interactome, cellular distribution, and substrate specificity. Our work demonstrates that the C-terminal region of TENT5D is both necessary and sufficient to dictate its ER targeting, FNDC3 binding, and effects on secretory proteins. Indeed, grafting the TENT5D C-terminal tail onto the cytosolic TENT5B is sufficient to reprogram its localization and function, while removing it from TENT5D abolishes its impact on the secretory proteome. These findings demonstrate that the C-terminal sequence can act as a molecular “zip code” to direct RNA-stabilizing enzymes to distinct subcellular compartments and physiological roles. In line with this model, our proteomic data show that the expression of ER-localized TENT5D, despite its lower protein stability, can coordinately boost the expression of hundreds of proteins across secretory organelles, selectively enhancing multiple components of the UPR, ERAD, and ER-to-Golgi transport. The integration of RNA-seq and TAIL-seq analyses further demonstrates that this remodeling is accompanied by the widespread elongation of poly(A) tails and increased abundance of transcripts encoding secretory pathway components, providing direct evidence for a polyadenylation-dependent mechanism. This degree of organellar reprogramming by a single RNA-modifying enzyme highlights the potency of localized mRNA regulation and reinforces the concept that ER-associated TENT5s are master regulators of secretory identity.^4^ In contrast, the role of TENT5B remains enigmatic. Despite a conserved catalytic core, TENT5B fails to localize to the ER and has a negligible impact on secretory gene expression, while it preferentially modulates proteins involved in cell cycle progression and division. This divergence suggests that TENT5B operates in a non-secretory context, consistent with prior associations with centrosome function and stemness.^18^^,^^19^

Mechanistically, our work reveals that FNDC3A and FNDC3B serve as key ER-anchoring factors for TENT5 enzymes. Silencing FNDC3s disrupts TENT5D’s ER association and reduces its ability to promote secretory output, establishing a direct link between intracellular localization and functional specificity. In addition, we found that TENT5 activity is further regulated by the HD, which contributes to protein stability. Our analysis of MM-associated mutations in TENT5C showed that nonsense mutations in the C-terminal tail eliminate ER targeting, while missense mutations in the HD significantly destabilize the protein, leading to its rapid proteasomal degradation. Both classes of mutations compromise Ig production and reduce cellular toxicity, mechanistically linking the reduction of antibody production with MM fitness and growth. These findings align with our previous hypothesis that transformed PCs may inactivate TENT5C to reduce the stress of Ig production and favor proliferation.^12^ Our current results now provide the molecular basis for this selective pressure, identifying the FNDC3-interaction region and HD as key regulatory modules in addition to the catalytic domain.

An important unresolved question concerns the degree of transcript specificity underlying ER-associated TENT5 activity. The integration of RNA-seq and proteomic datasets revealed a strong concordance between mRNA and protein changes, with the consistent enrichment of components of the ER, ERGIC, Golgi, and lysosomal pathways. Notably, among the relatively small subset of transcripts that were not upregulated, we did not detect enrichment for specific functional categories. Together with the global poly(A) tail elongation observed by TAIL-seq, these findings suggest that TENT5 activity is largely driven by subcellular localization and broadly affects transcripts encoding proteins destined for the secretory pathway rather than acting on highly selective transcript subsets.

More broadly, our findings reveal that evolutionary divergence in non-catalytic regions of TENT5 proteins underlies broad functional plasticity. This architectural modularity supports a model in which conserved enzymatic cores are repurposed by variable domains to control RNA stability in compartment-specific ways and fulfill distinct physiological roles.

In perspective, these insights have potential implications for RNA-based therapeutics. The ability to direct a poly(A) polymerase to specific subcellular compartments via engineered domains opens possibilities for the design of synthetic RNA stability regulators. Such tools could enhance the expression and translational efficiency of therapeutic RNA-based drugs and vaccines.

In summary, our study defines the domain-level logic by which TENT5 proteins achieve spatial specificity and functional specialization. By coupling catalytic activity to localization via FNDC3 interaction and HD-mediated stability, TENT5 enzymes emerge as modular regulators of compartmentalized gene expression. This work not only provides a mechanistic framework for understanding TENT5-related diseases, including MM, but also highlights new opportunities to leverage localized RNA regulation in therapeutic contexts.

### Limitations of the study

This study defines the molecular determinants that govern the ER recruitment of TENT5 proteins and their impact on the secretory proteome. However, some limitations should be considered. First, most mechanistic experiments were performed in cultured cell lines, which allowed controlled manipulation of TENT5 proteins and systematic mutational analysis. Although we complemented these findings with experiments in primary PCs and differentiation models, future *in vivo* studies will be required to fully define the physiological role of the TENT5–FNDC3 axis in tissue contexts. Second, while our integrated RNA-seq and proteomic analyses support a model in which ER-localized TENT5 proteins broadly stabilize transcripts encoding secretory pathway components, the precise molecular features that determine transcript susceptibility to TENT5-mediated polyadenylation remain unclear. Our data suggest that ER localization and signal peptide-dependent translation are major determinants, but additional layers of regulation may exist.

Finally, although we demonstrate that the C-terminal region of TENT5 proteins mediates ER targeting through FNDC3 interaction, the structural basis of this interaction and its regulation under physiological conditions remain to be elucidated. Future structural and biochemical studies will be needed to define how FNDC3 proteins recognize TENT5 C-terminal motifs and whether additional cofactors contribute to this process.

## Resource availability

### Lead contact

Further information and requests for resources and reagents should be directed to and will be fulfilled by the Lead Contact, Dr. Enrico Milan (milan.enrico@hsr.it).

### Materials availability

All unique/stable reagents generated in this study are available from the lead contact, Dr. Enrico Milan, upon request.

### Data and code availability

• Data: Processed results from proteomic and sequencing datasets are included in this publication as supplemental Excel files. Omics raw data are available in Mendeley Data doi: https://doi.org/10.17632/63fs23dx2g.1. RNA-seq and TAIL-seq raw data are available in GEO (GSE326742 and GSE326755, respectively). Other data are available upon request from the lead contact, Dr. Enrico Milan, upon request.
• Code: This paper does not report original code.

## Acknowledgments

We are particularly grateful to: Roberta Colzani for administrative assistance, Giovanni Tonon and Ineke Braakman for cells and antibodies, and all the Cenci Lab members for fruitful discussions.

Proteomics data on LP1 cells have been generated by the IFOM ETS Cogentech Proteomics and Metabolomics Core Facility (RRID:SCR_026937). Proteomics data on HEK293T cells have been generated by the ProMeFa facility, Ospedale San Raffaele. RNA sequencing was performed by Azenta Life Sciences. TAIL-seq analysis was performed by CD Genomics. The graphical abstract was created using BioRender.com. The work was supported by grants to E.M. from Fondazione Telethon and Fondazione Cariplo Joint grant on rare diseases (GJC21079/2022-0577), to S.C. from 10.13039/100020581Fondazione AIRC (Investigator Grant IG 2024 - 30332), to T.P. Fondazione Cariplo Young Researcher Grant (2023-1298); and to S.C. and E.M. from the European Union – Next Generation EU (National Recovery and Resilience Plan, Investment Partenariato Esteso PE8 “Conseguenze e sfide dell’invecchiamento,” Project Age-It - Aging Well in an Aging Society).

## Author contributions

L.V., D.L., S.P., M.V.M., and E.M. performed the experiments and analyzed the data; E.M. designed the research; U.O., L.G., and T.P. contributed with crucial methodologies and resources; T.P., S.C., and E.M. wrote the paper; T.P., S.C., and E.M. provided the research funds; S.C. and E.M. supervised the research.

## Declaration of interests

The authors declare no competing interests.

## Declaration of generative AI and AI-assisted technologies in the writing process

During the preparation of this work, the authors used ChatGPT in order to review and improve the English language. After using this tool, the authors reviewed and edited the content as needed and take full responsibility for the content of the publication.

## STAR★Methods

### Key resources table

**Table:** 

REAGENT or RESOURCE	SOURCE	IDENTIFIER
**Antibodies**		

mouse anti FLAG M2 antibody	Sigma-Aldrich	Cat# F1804; RRID:AB_262044
rabbit anti-FNDC3A	Novus Biological	Cat# NBP1-88809; RRID:AB_11024611
rabbit anti-FNDC3B	Novus Biological	Cat# NBP1-90495; RRID:AB_11034225
rabbit anti-FNDC3B	Abcam	Cat# ab135714
mouse anti-β-actin	Sigma-Aldrich	Cat# A5441; RRID:AB_476744
rabbit anti Calnexin/CNX	Proteintech	Cat# 10427-2-AP; RRID:AB_2069033
rabbit anti-lambda light chains	Dako	Cat# A0194
rabbit anti-LC3B	Novus Biological	Cat# NB100-2220; RRID:AB_10003146
rabbit anti-LDHA	Cell Signaling Technology	Cat# 3582; RRID:AB_2066887
rabbit anti-PDI	Dr. Ineke Braakman Lab, Utrecht, NL	N/A
mouse anti-PDI	Invitrogen	Cat# MA3-019; RRID:AB_2163120
rabbit anti-PRDX4	Proteintech	Cat# 10703-1-AP, RRID:AB_2168493
mouse anti-Ubiquitin monoclonal Ab (P4D1)	Santa Cruz Biotechnologies	Cat# sc-8017; RRID:AB_2762364
rabbit anti-TENT5C/FAM46C	Proteintech	Cat# 25038-1-AP, RRID:AB_2879863
rabbit anti-DYKDDDDK	Cell Signaling Technology	Cat# 14793; RRID:AB_2572291
FITC anti-CD271	BioLegend	Cat# 345104; RRID:AB_2282828
Alexa Fluor 647 goat anti-mouse IgG	Life Technologies	Cat# A21236; RRID:AB_2535805
Alexa Fluor 647 goat anti-rabbit IgG	Life Technologies	Cat# A21245; RRID:AB_2535813
Anti-rabbit IgG, HRP-linked	Cell Signaling Technology	Cat# 7074; RRID:AB_2099233
Anti-mouse IgG, HRP-linked	Cell Signaling Technology	Cat# 7076; RRID:AB_330924

**Chemicals, Peptides, and Recombinant Proteins**		

Bortezomib	Cell Signaling Technologies	Cat# 2204
Bafilomycin-A1	Cayman Chemical	Cat# 11038
Trypan Blue dye 0.40%	Bio-Rad	Cat# 1450013
Dimethyl sulphoxide	VWR Life Science	Cat# 0231-500ML
Protease inhibitor cocktail	Roche	Cat# 05056489001
Igepal CA-630	Sigma-Aldrich	Cat# I3021
Protein G Agarose beads	Millipore	Cat# 16-266
TriFAST	Euroclone	Cat# EMR507100
iTaq Univer SYBR Green Supermix	Bio-Rad	Cat# 1725122
DTT	Sigma-Aldrich	Cat# D9779
Iodoacetamide	Sigma-Aldrich	Cat# I1149
Trypsin sequencing grade	Roche	Cat# 11418475001
Quick Coomassie Stain	*Neo* Biotech	Cat# NB-45-00078
C18 Stage Tips	Proxeon Biosystems	Cat# SP301

**Critical Commercial Assays**		

DC protein assay	Bio-Rad	Cat# 5000116
Qproteome Cell compartment kit	Qiagen	Cat# 37502
5× In-Fusion Snap Assembly Master Mix	Takara Bio	Cat# ST2320
PrimeScriptTM RT reagent Kit with gDNA Eraser	Takara Bio	Cat# RR047A

**Deposited Data**		

RNA-seq	GEO	GSE326742
TAIL-seq	GEO	GSE326755
Omics Raw Data	Mendeley Data	doi: https://doi.org/10.17632/63fs23dx2g.1

**Experimental Models: Cell Lines**		

HEK293T	ATCC	RRID: CVCL_0063
HeLa	ATCC	RRID: CVCL_0030
LP1	Dr. Giovanni Tonon Lab, San Raffaele Scientific Institute	RRID: CVCL_0012

**Oligonucleotides**		

See qRT-PCR section for primer sequences		N/A

**Recombinant DNA**		

pLKO.2-puromycin Non-Mammalian shRNA	Sigma-Aldrich	SHC202
pLKO.2-puro shFNDC3B	Sigma-Aldrich	TRCN0000229812
pLKO.2-tGFP Non-Mammalian shRNA control	This paper, generated from Sigma-Aldrich SHC202	N/A
pLKO.2-tGFP shFNDC3A	Fucci et al., generated from Sigma-Aldrich TRCN0000338554	N/A
Human C-term FLAG-FAM46A cDNA	Genscript	OHu17426D
Human C-term FLAG-FAM46B cDNA	Genscript	OHu17470D
Human C-term FLAG-FAM46C cDNA	Genscript	OHu30151D
Human C-term FLAG-FAM46D cDNA	Genscript	OHu19029D
PGK-FLAG-TENT5 mutant/forms cDNAs-CD271	This Paper	N/A

**Software and Algorithms**		

ImageJ software	NIH	http://imagej.net/ij/
Bio-Rad CFX Maestro	Bio-Rad	https://www.bio-rad.com/it-it/product/cfx-maestro-software-for-cfx-real-time-pcr-instruments?ID=OKZP7E15
MaxQuant 2.0.3.0	Max Planck Institute of Biochemistry	https://www.maxquant.org/
Spectronaut v. 19.9	Biognosys	https://biognosys.com/software/spectronaut/
ClueGO	Cytoscape	https://cytoscape.org/
Prism v10.3.1	GraphPad	https://www.graphpad.com/scientific-software/prism/
NovaSeq Control Software v1.8.1	Illumina	https://support.illumina.com/downloads/novaseq-control-software-v1-8.html
Trimmomatic v.0.36	Usadel Lab	https://github.com/usadellab/Trimmomatic/releases
STAR aligner v.2.5.2 b	Cold Spring Harbor Laboratory	https://github.com/alexdobin/STAR
Perseus ver. 2.0.10.0	Max Planck Institute of Biochemistry	https://www.maxquant.org/perseus/

### Experimental model and study participant details

Human MM LP1 cells (female) were kindly provided by Dr. Giovanni Tonon, San Raffaele Scientific Institute. LP1 were genotyped as described in^23^ and with multiplex STR sequencing using PowerPlex 16 HS System (Promega, DC2101). Cells were periodically tested and confirmed negative for mycoplasma negativity. HEK293T (female) and HeLa (female) cells were purchased by ATCC.

Human PCs were obtained after written informed consent for research use, in accordance with the Declaration of Helsinki. Sample collection and use were approved by the local ethics committee (EMABANK- v.5, June 5, 2025; Comitato Etico Territoriale Lombardia 1, Via Olgettina 60, 20132 Milan, Italy). No experimental group allocation or stratification was performed. Information regarding ancestry, race or ethnicity was not collected. The study was not designed to assess sex-associated biological differences.

Primary murine PCs were obtained from both male and female 4–6 months old C57BL/6 wild-type mice (Charles River). Animals were housed and maintained under standard specific pathogen-free conditions at the San Raffaele Scientific Institute animal facility. Animal care and procedures were approved by the Italian Ministry of Health and the Institutional Animal Care and Use Committee Office (IACUC) of San Raffaele Scientific Institute under protocol #1163, in accordance with national and institutional guidelines for animal welfare.

### Method details

#### Cell culture

LP1 cells were cultured in RPMI media (Gibco-Life Technologies, 61870) supplemented 10% fetal bovine serum (S.I.A.L. group, SIALFBS-SA), L-glutamine (2 mM; Gibco-Life Technologies, 25030-024), penicillin (100 U/mL; Gibco-Life Technologies, 15140-122), streptomycin (100 μg/mL; Lonza), sodium pyruvate (1 mM; Gibco-Life Technologies, 11360-039). HEK293T and HeLa cells were cultured in Dulbecco’s modified Eagle’s medium (DMEM, Gibco-Life Technologies, 41965039) supplemented as described above for RPMI media. Cell viability of LP1 cells was assessed by counting them upon Trypan Blue dye 0.40% (Bio-Rad, 1450013) staining with TC20 Automated cell counter (Bio-Rad) at the indicated days after lentiviral infection. LP1 were treated with Bortezomib (1 μM, Cell Signaling, 2204) or Bafilomycin A1 (Cayman Chemical, 11038). Human PCs were purified with CD138 microbeads (Miltenyi Biotec, 130-097-614) following manufacturer’s instructions. Primary murine PCs were obtained by LPS-stimulation of purified splenic B cells as previously described in.^24^ Briefly B cells were purified from total splenocytes by immunomagnetic negative selection (130-090-862; Miltenyi Biotec), after homogenization and 7 min of hypotonic lysis of red cells (ammonium chloride–potassium bicarbonate buffer) and were cultured in RPMI medium supplemented as described above and with 2-mercaptoethanol (50 μM). Cells were activated with LPS (20 μg/mL) from Escherichia coli (strain 055:B55; L2880; Sigma).

#### Genetic manipulation

Human C-terminal FLAG-TENT5 cDNAs were purchased by GenScript (TENT5A: OHu17426D; TENT5B: OHu17470D; TENT5C: OHu30151D; TENT5D: OHu19029D) and cloned in a plasmid with a hybrid bidirectional human PGK-miniCMV promoter co-expressing the protein of interest and truncated human CD271. Truncated forms and MM-derived mutants were generated by specific PCRs followed by ligations, while TENT5D/TENT5B chimeras by homology recombination using 5× In-Fusion Snap Assembly Master Mix (Takara Bio, ST2320). Lentiviral viruses to stably express anti-FNDC3A (pLKO.2-tGFP shFNDC3A; TRCN0000338554), anti-FNDC3B (pLKO.2-puromycin shFNDC3B; TRCN0000229812) and control shRNAs (SHC202-puromycin and SHC202 tGFP) were generated starting from Mission shRNAs (Sigma-Aldrich) as described in.^11^ Lentiviral vectors were packaged with FLAG-proteins cDNA, pMD2-VSV-G, pMDLg/pRRE and pCMV-Rev plasmids in HEK293T cells for 14 h, then medium was replaced. 30 h after medium change cell supernatants were collected, ultra-centrifuged, filtered and added to LP1, HEK293T or HeLa cells. Lentiviral transduction was assessed checking CD271 expression with a FITC-conjugated anti-CD271 (BioLegend, 345104) using CytoFLEX S (Beckman Coulter). Plasmids used are listed in the key resources table.

#### Immunoblot analyses

Total protein extracts were obtained from LP1 cells by lysis in 150 mM NaCl, 10 mM Tris-HCl (pH 7.5), protease inhibitor cocktail (Roche, 05056489001) and 1% SDS (Sigma-Aldrich, 05030). Genomic DNA was mechanically removed using 0.5mL Insumed syringes (PIC solution). Protein amounts were quantified by DC protein assay (Bio-Rad, 5000116) as described by manufacturer. Western blots were performed using 10–20 μg of protein lysate in homemade (8%–15%) SDS-PAGE gels. Images were obtained using Uvitec Imager Mini HD9 (Uvitec Ltd) for HRP-conjugated secondary Ab or FLA9000 (FujiFilm) for Alexa Fluor conjugated secondary antibodies. Densitometric analysis was performed using ImageJ software (http://imagej.net/ij/). Antibodies used are listed in the key resources table. Cellular fractionations were performed with Qproteome cell compartment kit (Qiagen, 37502) following manufacturer’s instructions. In experiments analyzing cell compartment distribution, samples were loaded on a cell number basis.

#### Immunoprecipitation

Cells were lysed in 150 mM NaCl, 10 mM Tris-HCl (pH 7.5) and 1% Igepal CA-630 (Sigma-Aldrich, I3021), supplemented with protease inhibitors as described above, incubated for 15 min on ice. Nuclei and insoluble materials were pelleted at 1000 g for 15 min. For FLAG-TENT5 immunoprecipitation, soluble material was quantified, and 3–5 mg protein lysate was incubated for 16 h with 30 μL of protein G Agarose beads (Millipore, 16–266) pre-conjugated with 5 μg of mouse monoclonal FLAG M2 antibody (Sigma-Aldrich, F1804). Beads were washed 4 times in 150 mM NaCl, 10 mM Tris-HCl (pH 7.5), and resuspended in Laemmli Buffer for SDS-PAGE resolution.

#### Immunofluorescence

HeLa cells were seeded on slides, fixed with 4% paraformaldehyde, and permeabilized with PBS 0.1% Triton X-100 (Sigma-Aldrich, ×100). Cells were stained rabbit anti-DYKDDDDK (1:100; Cell Signaling Technology, 14793) and mouse anti-PDI (1:200; Invitrogen, MA3-019) washed in PBS, and stained with different Alexa Fluor 488 anti-mouse IgG (1:200; Life Technologies, A11029) Alexa Fluor 546 goat anti-rabbit IgG (1:200; Life Technologies, A11010), and DAPI (Sigma-Aldrich, MDB0020). Images were collected with Leica TCS SP8 confocal microscope using a 63× objective.

#### qRT-PCR

Total RNA was extracted from LP1 cells and murine PCs by lyses in TriFAST (Euroclone, EMR507100). 1000 ng of RNA were retro-transcribed with PrimeScriptTM RT reagent Kit with gDNA Eraser (Takara Bio, RR047A), following manufacturer’s instructions. cDNA corresponding to 5 ng of original RNA was used as template in qPCR reactions. qPCRs were performed using iTaq SYBR Green Supermix (Bio-Rad, 1725122) on Bio-Rad CFX96 PCR. Data were analyzed on Bio-Rad CFX Maestro software using H3 as normalizer.

Human primers used were:

BiP/HSPA5: FW TAGCGTATGGTGCTGCTGTC; REV TGACACCTCCCACAGTTTCA;

Ig LC: FW CAAGCCAACAAGGCCACACTA; REV GTCTCCACTCCCGCCTTGAC;

H3: FW GTGAAGAAACCTCATCGTTACAGGCCTGGT; REV CTGCAAAGCACCGATAGCTGCGCTCTGGAA;

PRDX4: FW AACAGCTGTGATCGATGGAG; REV TCAAGTCTGTCGCCAAAAGC;

TENT5C: FW CATTCGGCGTCAGTTTGAGT; REV TTCTTGGTGGCGATCAGTCT.

TENT5D: FW AGTTCACTCAGACGGCAGTT; REV TGCAGTACTTCAGAAGGCCA.

Murine primers used were:

FNDC3A: FW GGAGTTACTTGGCTGTCCCT; REV CACCACTTCGCTAGGATTGC

FNDC3B: FW CTCCAACCAGACCCCTCATT; REV GTGCCTGGTTTCAAATGGGT

H3: FW GTGAAGAAACCTCATCGTTACAGGCCTGGT; REV CTGCAAAGCACCAATAGCTGCACTCTGGAA

TENT5C: FW TCACCTCCTCTTCCAACGCC; REV AGGTTGGAAAGTTGCCTCGC

#### RNA library preparation, NovaSeq sequencing and data analysis

Total RNA was extracted from LP1 cells by lyses in TriFAST (Euroclone, EMR507100). DNA was removed with RNase-free DNase set (Qiagen, 79254) using RNeasy Mini Kit (Qiagen, 74104). RNA-sequencing was performed by Azenta Life Sciences. RNA samples were quantified using Qubit 4.0 Fluorometer (Life Technologies, Carlsbad, CA, USA) and RNA integrity was checked with RNA Kit on Agilent 5300 Fragment Analyzer (Agilent Technologies, Palo Alto, CA, USA). RNA sequencing libraries were prepared using the NEBNext Ultra II RNA Library Prep Kit for Illumina following manufacturer’s instructions (NEB, Ipswich, MA, USA). Briefly, mRNAs were first enriched with Oligo(dT) beads. Enriched mRNAs were fragmented according to manufacturer’s instruction. First strand and second strand cDNAs were subsequently synthesized. cDNA fragments were end repaired and adenylated at 3′ends, and universal adapters were ligated to cDNA fragments, followed by index addition and library enrichment by limited-cycle PCR. Sequencing libraries were validated using NGS Kit on the Agilent 5300 Fragment Analyzer (Agilent Technologies, Palo Alto, CA, USA), and quantified by using Qubit 4.0 Fluorometer (Invitrogen, Carlsbad, CA). The sequencing libraries were multiplexed and loaded on the flow cell on the Illumina NovaSeq X plus instrument according to manufacturer’s instructions. The samples were sequenced using a 2 × 150 Pair-End (PE) configuration v1.5. Image analysis and base calling were conducted by the NovaSeq Control Software v1.8.1 on the NovaSeq instrument. Raw sequence data (.bcl files) generated from Illumina NovaSeq was converted into fastq files and de-multiplexed using Illumina bcl2fastq program version 2.20. One mismatch was allowed for index sequence identification. After investigating the quality of the raw data, sequence reads were trimmed to remove possible adapter sequences and nucleotides with poor quality using Trimmomatic v.0.36. The trimmed reads were mapped to the reference genome available on ENSEMBL using the STAR aligner v.2.5.2 b. BAM files were generated as a result of this step. Unique gene hit counts were calculated by using feature Counts from the Subread package v.1.5.2. Only unique reads that fell within exon regions were counted. After extraction of gene hit counts, the gene hit counts table was used for downstream differential expression analysis. Using DESeq2, a comparison of gene expression between the groups of samples was performed. The Wald test was used to generate *p* values and Log2 fold changes. Genes with adjusted *p* values <0.05 and absolute log2 fold changes >1 were called as differentially expressed genes for each comparison.

#### TAIL-seq

Total RNA was extracted from LP1 cells by lyses in TriFAST (Euroclone, EMR507100). DNA was removed with RNase-free DNase set (Qiagen, 79254) using RNeasy Mini Kit (Qiagen, 74104). TAIL-seq was performed by CD Genomics. TAIL Iso-seq libraries were constructed using the PCR-cDNA Barcoding Kit (Oxford Nanopore Technologies, SQK-PCB114.24) according to the manufacturer’s protocol. Briefly, 250 ng of high-quality total RNA was adjusted to a final volume of 10 μL with nuclease-free water. Reverse transcription (RT) primers were annealed to the RNA templates, and the complexes were purified using RNA Clean magnetic beads. First-strand cDNA synthesis was then conducted, and the resulting products were subjected to PCR amplification. The amplified cDNA was subsequently purified using AMPure XP beads (Beckman Coulter). Following purification, sequencing adapters were attached to the amplified cDNA to complete the library preparation. The final libraries were loaded onto an R10.4.1 flow cell and sequenced on a PromethION platform (ONT) for 48–72 h.

#### Mass spectrometry analysis

Immunoprecipitation of FLAG-TENT5B and TENT5D was performed in LP1 cells with mouse monoclonal FLAG M2 antibody (Sigma-Aldrich, F1804) as described in the dedicated section. For label-free analysis of TENT5 effects, TENT5B and TENT5D overexpressing and mock LP1 and HEK203T cells were collected 5 days after infection. Cells were lysed in 150 mM NaCl, 10 mM Tris-HCl (pH 7.5), protease inhibitor cocktail (Roche, 05056480001) and 1% SDS (Sigma-Aldrich, 05030). Genomic DNA was mechanically removed using 0.5mL Insumed syringes (PIC solution). Immunoprecipitated proteins or 55 μg of total protein lysate were loaded in a 4–12% gradient SDS-PAGE and stained with Quick Coomassie Stain (*Neo* Biotech, NB-45-00078). Gel slices sampling the entire length of the lanes were excised and processed as described in.^25^ Briefly, after reduction with 10mM DTT (Sigma-Aldrich, D9779), alkylation with 55 mM iodoacetamide (Sigma-Aldrich, I1149) and overnight digestion with trypsin 10ng/μL (Roche, 11418475001), tryptic peptides were desalted and concentrated on C18 Stage Tips (Proxeon Biosystems, SP301) following the manufacturer’s instructions and analyzed by LC-MS/MS.

For the IPs experiment, each digested sample was analyzed as a technical replicate on the Vanquish *Neo* nLC chromatographic system coupled with an Orbitrap Exploris 480, equipped with a Nano Flex ESI source and the FAIMS Pro interface operating at CV -50/-70 (Thermo Fisher Scientific). The nLC was connected to a 25 cm × 75 μm in-house packed n-column with ReproSil-Pur C18-AQ 1.9 μm beads. Peptides were separated using a linear gradient of 50 min, starting from 100% solvent A (2% ACN, 0.1% formic acid) and ramping to 12% solvent B (80% acetonitrile, 0.1% formic acid) in 3 min, then increasing to 50% B over 43 min, and reaching 100% B in 2 min. A washout step of 2 min at 100% B followed. The flow rate was maintained at 0.20 μL/min. MS data were acquired in positive DDA mode, with a top 28 method for HCD fragmentation. Full MS spectra (300–1000 Th) were acquired in the Orbitrap at 60 K resolution, with maximum IT mode set to 100 ms, normalized AGC target at 100%, Microscan 1, RF Lens at 40%. Fragment spectra were acquired with a resolution of 15 K, Normalized AGC target at 100%, Microscan 1, maximum IT set to Auto, with normalized collision energy at 28%, an isolation width of 1.6 m/z, and an exclusion duration of 20 s.

LP1 total lysate digested samples were analyzed using the nLC-MS/MS system described above. Peptide separation was performed at a flow rate of 0.20 μL/min on a linear gradient lasting 131 min. The gradient started with 97% solvent A for 1 min, then ramped to 19% solvent B over 72 min, followed by an increase to 29% B in 28 min, and then to 41% B in 20 min. A washout step was carried out for 10 min, reaching 95% B. MS data were acquired in positive DIA mode using an optimized MvM method.^26^ For full MS spectra (400–1000 Th), the resolution was set at 120 K, with an AGC Target Normalized of 300%, an IT of 55 ms, and 1 Microscan. For fragment spectra, the resolution was 30 K, AGC Target Normalized was 1000%, the IT was set to Auto, and 1 Microscan. Isolation windows of 30 m/z were used, with 20 scan events. The Normalized Collision Energy was set at 32%. HEK293T digested samples were identified using the nLC-MS/MS system and analyzed as described in.^27^

Data analysis for IPs experiment was carried out with MaxQuant 2.0.3.0, searching against the Uniprot_CP_Human_2020, setting Label Free quantitation, trypsin as enzyme and up to two missed cleavages. Cysteine carbamidomethyl was used as fixed modification, methionine oxidation and protein N-terminal acetylation as variable modifications. Mass deviation for MS-MS peaks was set at 20 ppm. The peptides and protein false FDR were set to 0.01; the minimal length required for a peptide was seven amino acids; a minimum of two peptides and at least one unique peptide were required for high-confidence protein identification. Perseus ver. 2.0.10.0,^28^ was used for statistical analysis, considering averaged Protein LFQ Intensities which were log2 transformed and Imputation was applied. Differential proteins were calculated applying *t* test and *p*-value 0,05; statistically significant proteins are represented in the volcano plots.

Raw data of the LP1 total lysate analysis were processed with Spectronaut v. 19.9 using slightly modified Biognosys default settings, in directDIA mode with iRT regression strategy and the Uniprot_CP_Human_2020 database. Briefly, trypsin was set as the digestion enzyme, and up to two missed cleavages were allowed. Cysteine carbamidomethylation was set as fixed modification, while Methionine Oxidation and Protein N-term Acetylation were set as variable modifications. Peptide and protein FDR were both set at 0.01. Quantification was based on MS2 area, applying cross-run normalization. Statistical analysis was performed with Perseus considering the protein MS2Quantity normalized based on the *Z* score; the function ‘imputation’ function was used to replace missing values from a normal distribution. Supervised Hierarchical Clustering analysis was done applying ANOVA with FDR 0.01. The heatmap represented statistically significant proteins. Proteins significantly modulated by TENT5 proteins in LP1 cells were run through functional annotation clustering on ClueGO (Cytoscape) using the list of quantified proteins as background, GOterm-BP-FAT as search parameters. In the HEK293T experiment, proteins quantified in all three biological replicates in at least one experimental group were first selected for the cell compartment analysis, and missing values were subsequently imputed using Perseus. Cell compartment analyses in LP1 and HEK293T cells were performed using the following categories: (Nucleus GO:0005634; Cytosol GO:0005829; Mitochondria GO:0005739; Ribosome GO: 0005840; Peroxisome GO:0005777; ER GO:0005783; ERGIC GO:0033116; Golgi GO:0005794; Lysosome GO:0005764).

### Quantification and statistical analysis

Graph generation and statistical analyses for non-omics experiments were performed using GraphPad Prism v10.3.1 (GraphPad Software). Figures were assembled using Adobe Illustrator. Data are presented as mean ± SEM unless otherwise indicated. Statistical details for each experiment are reported in the corresponding figure legends. Omics data analyses and related statistical methods are described in the dedicated method details subsections. *p* values < 0.05 were considered statistically significant. Asterisks indicate the following *p* values: ^∗^*p* < 0.05; ^∗∗^*p* < 0.01; ^∗∗∗^*p* < 0.001; ^∗∗∗∗^*p* < 0.0001.

## Supplemental information

Document S1. Figures S1–S6

Data S1. Label-free MS/MS analysis of TENT5D and TENT5B overexpressing LP1 cells, related to Figure 2

Data S2. Label-free MS/MS analysis of TENT5D and TENT5B overexpressing HEK 293T cells, related to Figure S2

Data S3. RNA-seq analysis of TENT5D and TENT5B overexpressing LP1 cells, related to Figure 2

Data S4. TAIL-seq analysis of TENT5D and TENT5B overexpressing LP1 cells, related to Figure 3

Data S5. MS/MS analysis of TENTD and TENT5B interactome in LP1 cells, related to Figure 3

Document S2. Article plus supplemental information
