# Supplementary material for: The C-terminal region of TENT5 proteins drives ER-associated mRNA polyadenylation via FNDC3 interaction
Source: Cell Rep. 2026 Jun 4;45(6):117501. doi: 10.1016/j.celrep.2026.117501 (PMC13291444; doi:10.1016/j.celrep.2026.117501)
Supplement: Document S1. Figures S1–S6 [file mmc1.pdf]

Cell Reports, Volume *45*

Supplemental information

The C-terminal region of TENT5 proteins drives ER-associated mRNA polyadenylation via FNDC3 interaction

Lisa Viviani, Daniel Lacidogna, Sara Pennacchio, Maria Vittoria Mengozzi, Ugo Orfanelli, Leone Giordano, Tommaso Perini, Simone Cenci, and Enrico Milan

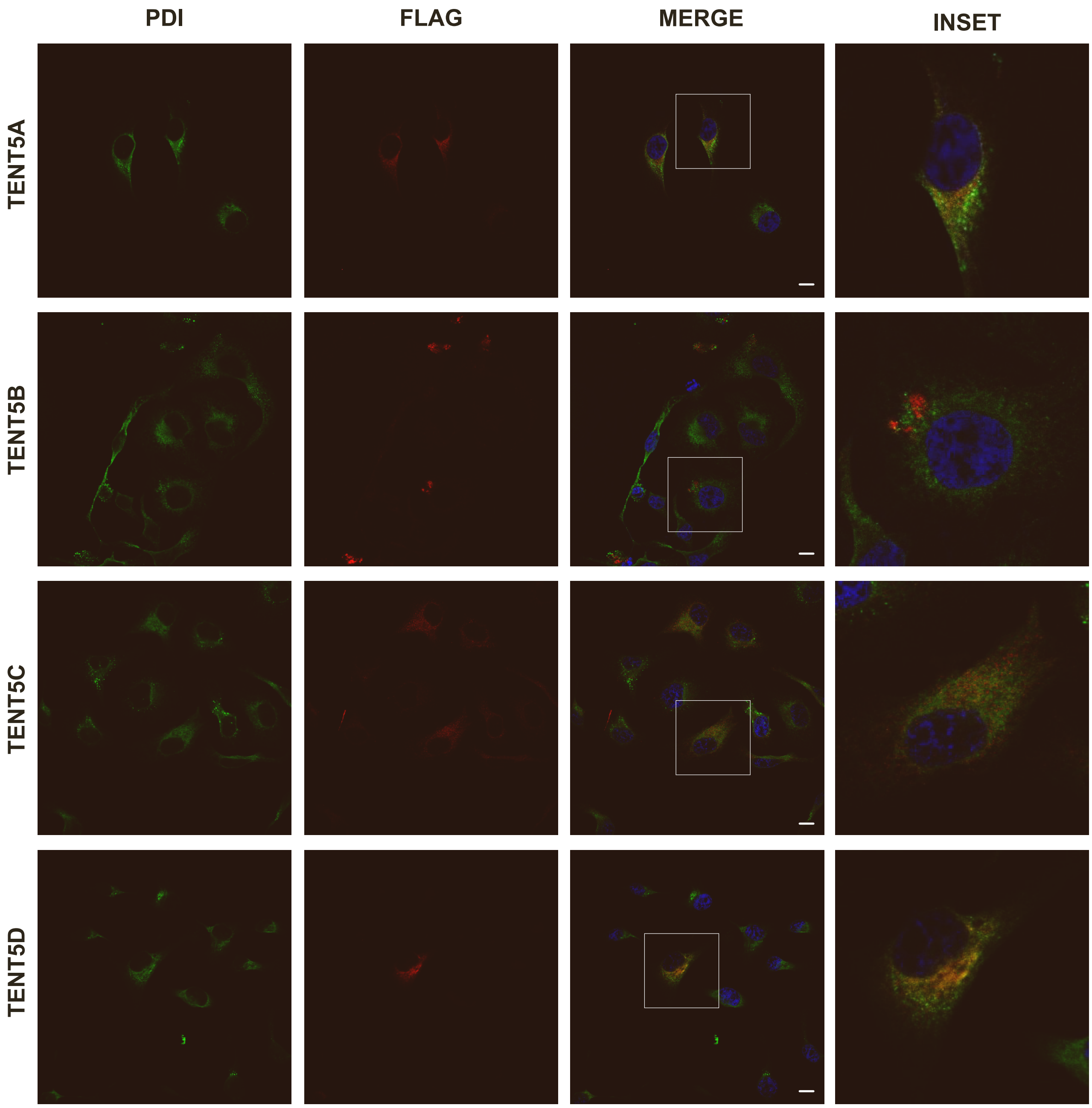

Figure S1. ER-associated TENT5 paralogs display distinct subcellular localization patterns. Related to Figure 1. Immunofluorescence analysis of FLAG-TENT5s (red) and PDI (green) in HeLa cells. Nuclei are stained blue with DAPI. White insets show detail magnification. Scale bar, 10 µm.

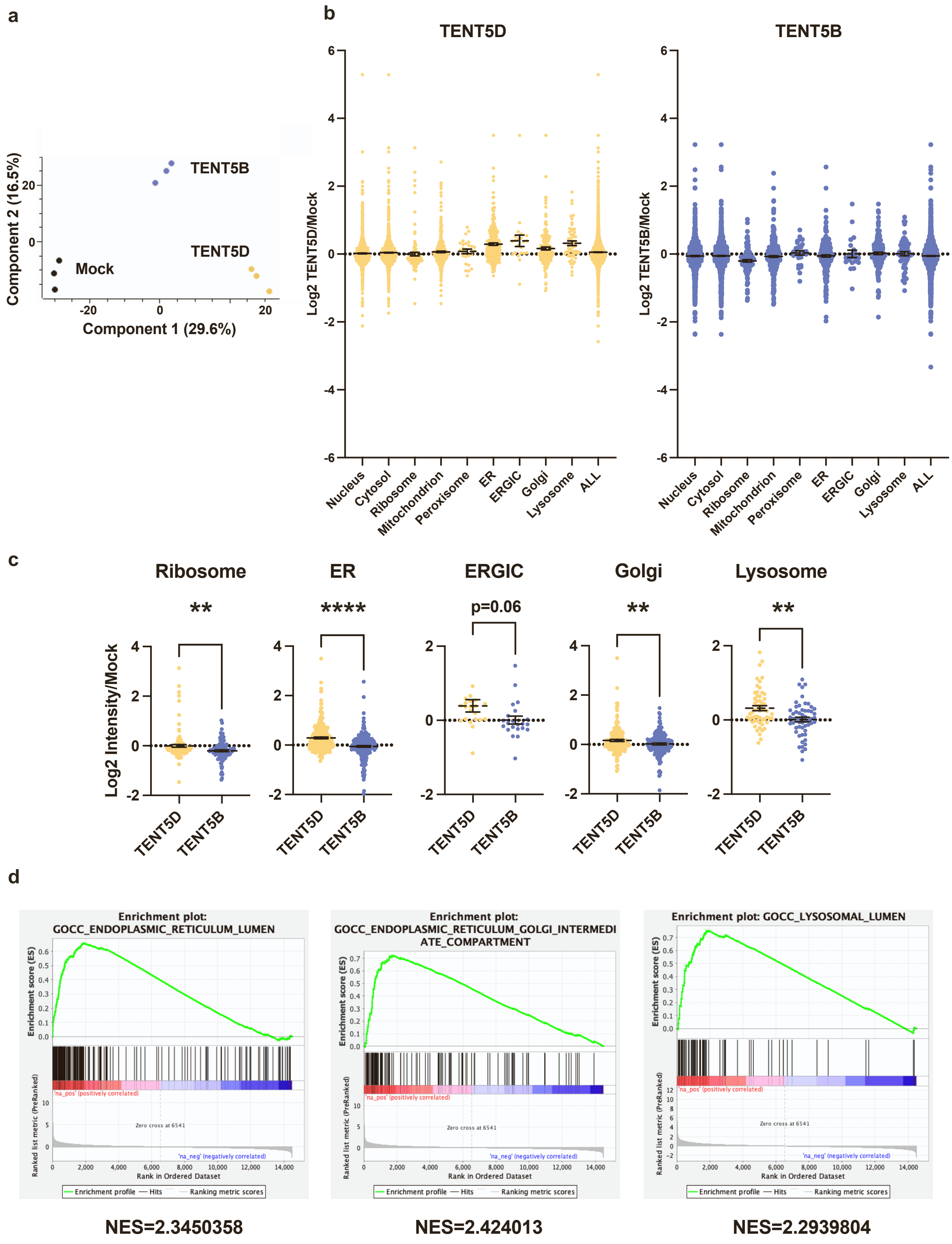

Figure S2. TENT5D extensively remodels secretory pathway proteomes and transcriptomes. Related to Figure 2. (a) Principal Component Analysis (PCA) of proteome changes in LP1 cells overexpressing TENT5D or TENT5B by label-free LC-MS/MS analysis. (**b**) Proteome changes in HEK293T cells overexpressing TENT5D (left) or TENT5B (right) compared to mock infected cells by label-free LC-MS/MS analysis. The 2606 proteins quantified in all the 3 biological replicate in at

least 1 experimental condition were grouped by the indicated GO categories (average ratios ± SEM). (**c**) Fold change relative to mock-infected HEK293T cells of quantified proteins belonging to the indicated cell compartments (average ratios ± SEM, unpaired t-test, ***P<0.01; ****P<0.0001). (**d**) GSEA of RNA-seq data of TENT5D expressing LP1 cells for the indicated GO CC terms compared to mock infected cells.

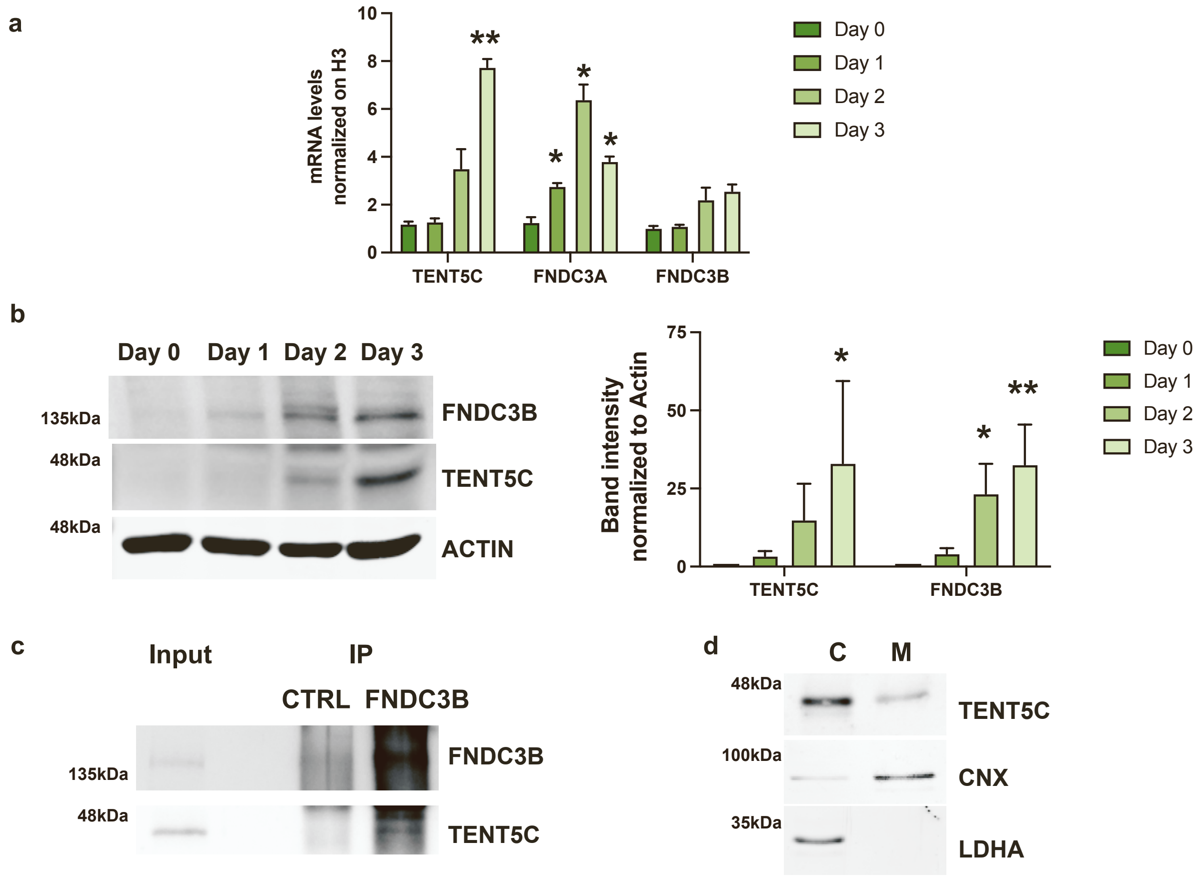

Figure S3. The TENT5–FNDC3 axis is engaged in normal and malignant plasma cells. Related to Figure 3. (**a**) qRT-PCR analysis of TENT5C, FNDC3A and FNDC3B mRNA levels in LPS-activated murine splenic B cells. (mean ± SEM normalized on H3 mRNA; Repeated Measure One-Way ANOVA with Dunnett's multiple comparison *vs* day 0, n=3, **P*<0.05; ***P*<0.01. (**b**) Analysis of TENT5C and FNDC3B protein levels in LPS-activated murine splenic B cells. Left: representative blots, right: quantification of protein band intensity normalized to actin expressed relative to day 0. (mean ± SEM, Kruskal Wallis test with Dunn's multiple comparison *vs* day 0, n=6, **P*<0.05; ***P*<0.01). (**c**) Co-immunoprecipitation of TENT5C with FNDC3B in human plasma cells purified form tonsil. (**d**) Immunoblot analysis for cellular distribution of TENT5C protein in patient derived MM cells (C= Cytosol, M= Membrane).

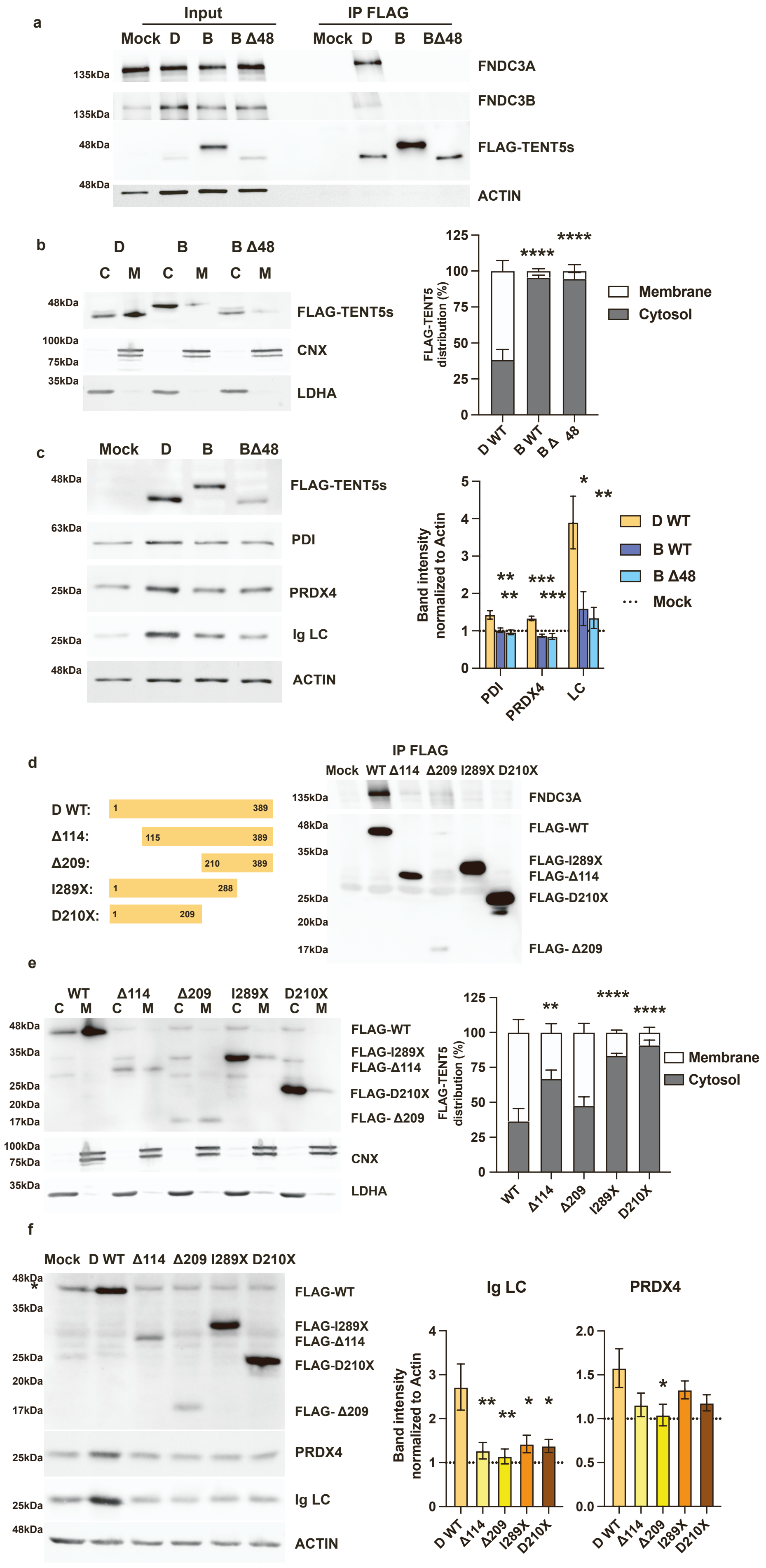
a
Input
IP FLAG
Mock D B B Δ48
Mock D B BΔ48
135kDa
135kDa
48kDa
48kDa
FNDC3A
FNDC3B
FLAG-TENT5s
ACTIN
b
D B B Δ48
C M C M C M
48kDa
100kDa
75kDa
35kDa
FLAG-TENT5s
CNX
LDHA
FLAG-TENT5 distribution (%)
0 25 50 75 100 125
D WT
B WT
B Δ 48
****
****
Membrane
Cytosol
c
Mock D B BΔ48
48kDa
63kDa
25kDa
25kDa
48kDa
FLAG-TENT5s
PDI
PRDX4
Ig LC
ACTIN
Band intensity normalized to Actin
0 1 2 3 4 5
PDI
PRDX4
LC
D WT
B WT
B Δ48
Mock
d
D WT: 1 389
Δ114: 115 389
Δ209: 210 389
I289X: 1 288
D210X: 1 209
IP FLAG
Mock WT Δ114 Δ209 I289X D210X
135kDa
48kDa
35kDa
25kDa
20kDa
17kDa
FNDC3A
FLAG-WT
FLAG-I289X
FLAG-Δ114
FLAG-D210X
FLAG- Δ209
e
WT Δ114 Δ209 I289X D210X
C M C M C M C M C M
48kDa
35kDa
25kDa
20kDa
17kDa
100kDa
75kDa
35kDa
FLAG-WT
FLAG-I289X
FLAG-Δ114
FLAG-D210X
FLAG- Δ209
CNX
LDHA
FLAG-TENT5 distribution (%)
0 25 50 75 100 125
WT
Δ114
Δ209
I289X
D210X
**
****
****
Membrane
Cytosol
f
Mock D WT Δ114 Δ209 I289X D210X
48kDa
35kDa
25kDa
20kDa
17kDa
25kDa
25kDa
48kDa
FLAG-WT
FLAG-I289X
FLAG-Δ114
FLAG-D210X
FLAG- Δ209
PRDX4
Ig LC
ACTIN
Ig LC
PRDX4
Band intensity normalized to Actin
0 1 2 3 4
0.0 0.5 1.0 1.5 2.0
D WT
Δ114
Δ209
I289X
D210X

Figure S4. The C-terminal region of TENT5D is required for ER localization and secretory activity. Related to Figure 4. (a) Co-immunoprecipitation of FNDC3 proteins with FLAG-TENT5D, TENT5B or its truncated form in LP1 cells. (**b**) Immunoblot analysis of the cellular distribution of TENT5D, TENT5B and its truncated form in LP1 cells. Left: representative blot; right: quantification of FLAG-TENT5 abundance in the cytosolic and membrane-associated fractions (mean ± SEM; Ordinary Two-way ANOVA with Dunnett's multiple comparison *vs* TENT5D-WT infected cells, n= 3, ****P<0.0001). **(c)** Immunoblot analysis of the indicated proteins in LP1 cells expressing TENT5D, TENT5B and its truncated form. Left: representative blots, right: quantification of protein band intensity normalized to actin, expressed as relative to mock-infected cells (mean ± SEM, Ordinary One-Way ANOVA with Dunnett's multiple comparisons *vs* TENT5D-WT, n=5, *P<0.05; **P<0.01, ***P<0.001). (**d**) Co-immunoprecipitation of FNDC3 proteins with FLAG-TENT5D and its truncated forms in LP1 cells. Left: schematic representations of TENT5D truncated forms, right: Representative immunoblots. (**e**) Immunoblot analysis of the cellular distribution of TENT5D truncated forms in LP1 cells. Left: representative blot; right: quantification of FLAG-TENT5 abundance in the cytosolic and membrane-associated fractions (mean ± SEM; Ordinary Two-way ANOVA with Dunnett's multiple comparison *vs* TENT5D-WT infected cells, n= 5, **P<0.01; ****P<0.0001). (**f**) Immunoblot analysis of the indicated proteins in LP1 cells expressing TENT5D and its truncated forms. Left: representative blots (* = aspecific band), right: quantification of protein band intensity normalized to actin, expressed as relative to mock-infected cells (mean ± SEM, Ordinary One-Way ANOVA with Dunnett's multiple comparisons *vs* TENT5D-WT, n=5, *P<0.05; **P<0.01).

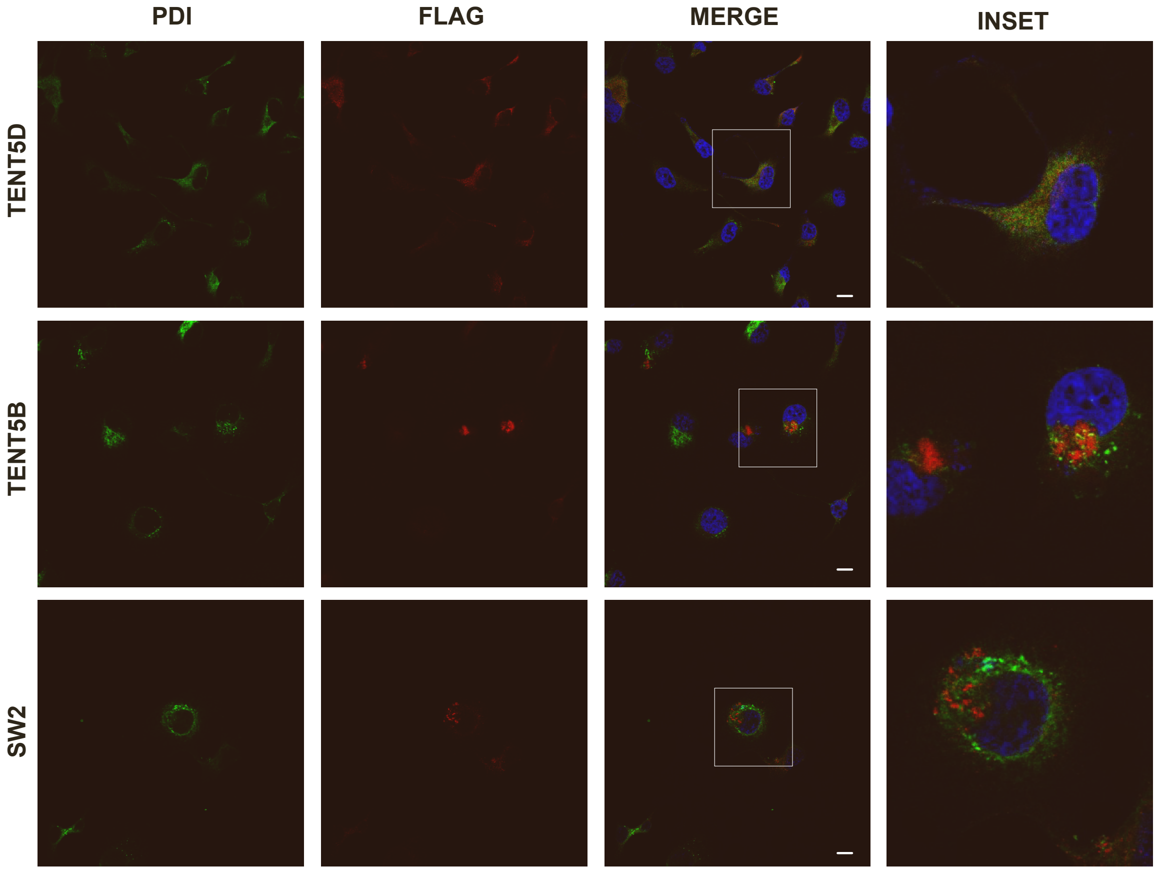

Figure S5. SW2 region controls TENT5D ER localization by confocal microscopy. Related to Figure 5. Immunofluorescence analysis of FLAG-TENT5D, TENT5B or SW2 mutant (red) and PDI (green) in HeLa cells. Nuclei are stained blue with DAPI. White insets show detail magnification. Scale bar, 10 µm.

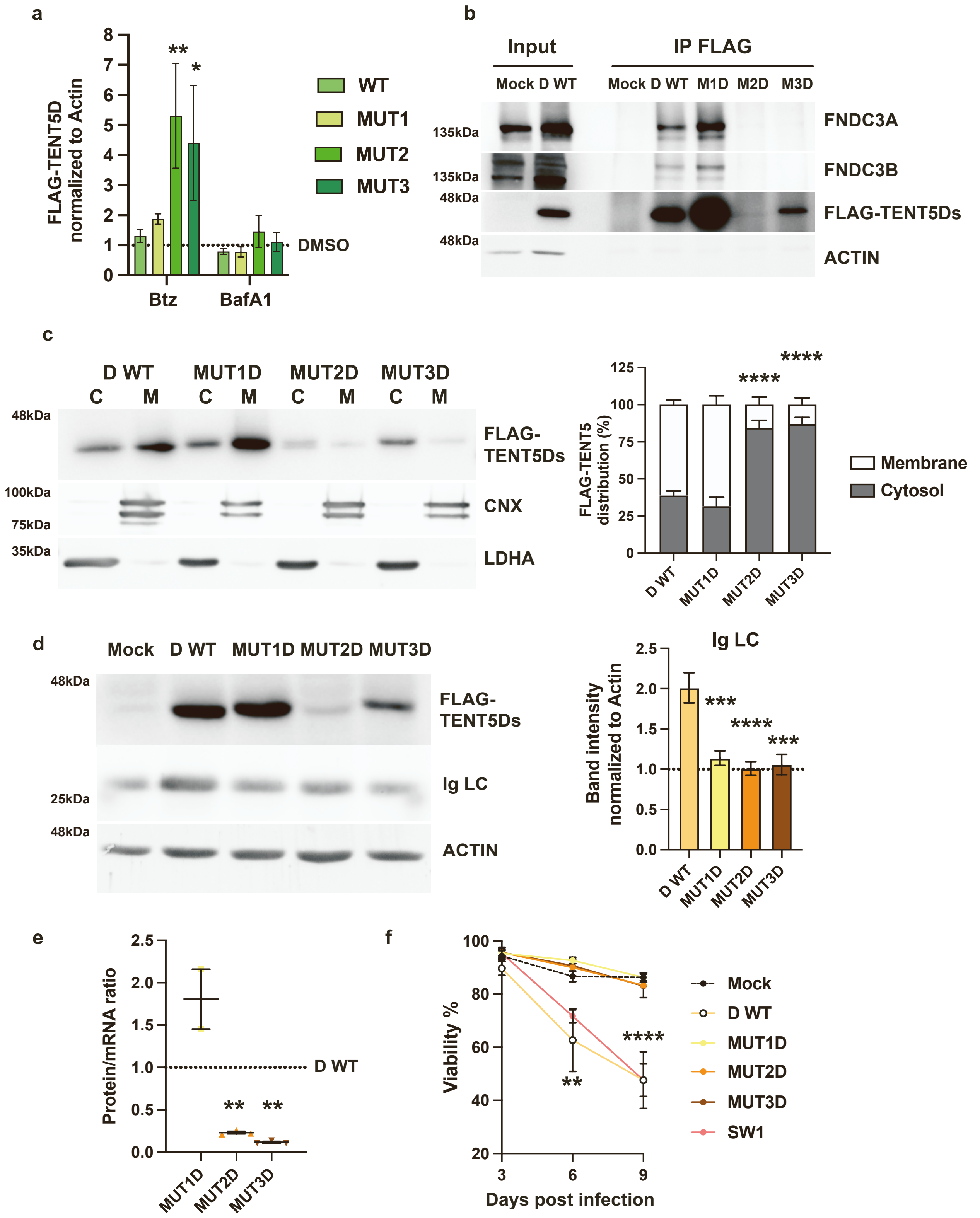

Figure S6. Multiple myeloma-derived TENT5 mutations impair protein stability and ER-associated functions. Related to Figure 6. (a) Quantification of protein band intensity normalized to actin in LP1 cells expressing TENT5C or its MM-derived mutants treated with Bortezomib (1µM) or Bafilomycin A1 100 nM for 5h (representative blot in **Figure 6d**, Fold changes relative to DMSO treated cells ± SEM, n=3, Kruskal Wallis test with Dunn's multiple comparison *vs* DMSO

treated cells, n=5, *P<0.05; **P<0.01). **(b)** Co-immunoprecipitation of FNDC3 proteins with FLAG-TENT5D or its MM-derived mutants in LP1 cells (**c**) Immunoblot analysis of the cellular distribution of TENT5D and its MM-derived mutants in LP1 cells. Left: representative blot; right: quantification of FLAG-TENT5 abundance in the cytosolic and membrane-associated fractions (mean ± SEM; Ordinary Two-way ANOVA with Dunnett's multiple comparison *vs* TENT5D-WT infected cells, n= 3, ****P<0.0001). (**d**) Immunoblot analysis of the indicated proteins in LP1 cells expressing TENT5D or its mutants. Left: representative blots, right: quantification of protein band intensity normalized to actin (mean ± SEM, Ordinary One-Way ANOVA with Dunnett's multiple comparisons *vs* TENT5D-WT, n=6, ***P<0.001, ****P<0.0001). (**e**) Ratio between protein intensity measured by immunoblot and mRNA levels measured by RT-qPCR for TENT5D and its mutants in LP1 cells (mean ± SEM, n=3, Ordinary One-Way ANOVA with Dunnett's multiple comparisons *vs* TENT5D-WT, n=3, **P<0.01). (**f**) Cell Viability of LP1 expressing TENT5D or its mutants assessed by trypan blue staining after lentiviral infection (mean ± SEM, Ordinary Two-way ANOVA with Dunnett's multiple comparison *vs* mock cells, n=3, **P<0.01, ****P<0.0001).
